# Fungal diagnostics and antifungal drug access in Latin America and the Caribbean: an ESCMID EFISG multinational survey

**DOI:** 10.1038/s41467-026-73165-2

**Published:** 2026-07-28

**Authors:** Alessandro C. Pasqualotto, Indira Berrio, Bryan Ortiz, Julio Maquera-Afaray, Norma Fernández, Diana Varela, Luis E. Cuéllar, Freddy Pérez, Mayda Rosa Perurena Lancha, Diana Perales, Maribel Dolande, Alexandro Bonifaz, Andres Ceballos, Tulio C. O. Magalhães, Oliver A. Cornely, Diego R. Falci, Jon Salmanton-García

**Affiliations:** 1 Federal University of Health Sciences of Porto Alegre (UFCSPA), Porto Alegre, Brazil; 2 Santa Casa de Porto Alegre, Porto Alegre, Brazil; 3 European Fungal Infection Study Group, European Society of Clinical Microbiology and Infectious Diseases (EFISG/ESCMID), Basel, Switzerland; 4 Medical and Experimental Mycology Group, Corporacion para Investigaciones Biologicas (CIB), Medellin, Colombia; 5 Hospital General de Medellin Luz Castro de Gutierrez ESE, Medellin, Colombia; 6 Basic and Applied Microbiology Research Group (MICROBA), School of Microbiology, Universidad de Antioquia, Medellin, Colombia; 7 Instituto de Investigaciones en Microbiologia, Facultad de Ciencias, Universidad Nacional Autónoma de Honduras, Tegucigalpa, Honduras; 8 Universidad Nacional Daniel Alcides Carrion, Cerro de Pasco, Peru; 9 Hospital EsSalud II Pasco, Cerro de Pasco, Peru; 10 Laboratorio de Micologia, Hospital de Clinicas Jose de San Martin, Buenos Aires, Argentina; 11 Servicio de Infectologia, Servicio de Atención Integral de Pacientes con VIH, Hospital Escuela, Tegucigalpa, Honduras; 12 Instituto Hondureño de Enfermedades Infecciosas y Parasitología Antonio Vidal, Tegucigalpa, Honduras; 13 Instituto Nacional de Enfermedades Neoplasicas, Lima, Peru; 14 Universidad Nacional Federico Villarreal, Lima, Peru; 15 Head of Department, Instituto de Medicina Tropical “Pedro Kourí”, La Habana, Cuba; 16 Infectious Diseases Department, ISSSTE General Hospital, San Luis Potosi, Mexico; 17 Department of Mycology, Instituto Nacional de Higiene “Rafael Rangel”, Caracas, Venezuela; 18 Dermatology Service, Mycology Department, Hospital General de México “Dr. Eduardo Liceaga”, Mexico City, Mexico; 19 Infectious Diseases Research Group, Microbiology Department, Pontificia Universidad Javeriana, Bogotá, Colombia; 20 Hospital de Clínicas de Porto Alegre, Porto Alegre, Brazil; 21 Institute of Translational Research, Cologne Excellence Cluster on Cellular Stress Responses in Aging-Associated Diseases (CECAD), Faculty of Medicine, University of Cologne, Cologne, Germany; 22 Department I of Internal Medicine, Center for Integrated Oncology Aachen Bonn Cologne Duesseldorf (CIO ABCD) and Excellence Center for Medical Mycology (ECMM), Faculty of Medicine, University Hospital Cologne, University of Cologne, Cologne, Germany; 23 German Centre for Infection Research (DZIF), Partner Site Bonn-Cologne, Cologne, Germany; 24 Clinical Trials Centre Cologne (ZKS Köln), Faculty of Medicine, University of Cologne, Cologne, Germany

**Keywords:** Epidemiology, Outcomes research, Fungal infection

## Abstract

Invasive fungal diseases (IFD) pose a major health challenge in Latin America and the Caribbean (LAC), particularly in vulnerable populations. A cross-sectional, survey is distributed between April 2023 and May 2025 to institutions involved in IFD diagnosis or care across LAC. The questionnaire evaluates diagnostic tools, antifungal availability, and therapeutic drug monitoring (TDM). A total of 619 institutions from 23 countries across LAC participate. *Candida* spp. (92%) and *Aspergillus* spp. (54%) are most frequently reported as major fungal threats. Culture (90%) is widely available, whereas access to galactomannan (41%), β-D-glucan (29%), and molecular testing (23%) is considerably lower. Availability of antifungals, including liposomal amphotericin B (38%), echinocandins (51%), voriconazole (57%) or posaconazole (33%) is significantly higher in countries with GDP per capita >US$ 10,000, in transplant centres, and in institutions managing people living with HIV. Therapeutic drug monitoring is available in only 36% of centres. Major diagnostic and treatment gaps persist in low-income countries, particularly in access to tools for identifying endemic mycoses. Substantial disparities exist in IFD diagnostic and treatment capacity across LAC, primarily driven by national income and institutional complexity. Strengthening laboratory infrastructure, antifungal access, and integration of fungal disease management into public health systems is urgently needed.

## Abstract (web summary)

Here, in a survey of 619 institutions across Latin America and the Caribbean, the authors showcase major gaps in fungal diagnostics and antifungal access, with stark disparities linked to income and institutional capacity, underscoring the urgent need for equitable care.

## Introduction

Invasive fungal diseases (IFD) remain a major public health concern worldwide, with particularly severe impact in low- and middle-income countries, notably in Latin America and the Caribbean (LAC)^1,2^. LAC bears a high burden of neglected systemic mycoses, including coccidioidomycosis, histoplasmosis, and paracoccidioidomycosis, which disproportionately affect immunocompromised populations, such as people living with HIV/AIDS (PLWHA), cancer patients, individuals with tuberculosis (TB), or solid organ transplant (SOT) recipients^2–4^. Despite increased attention in global health, many LAC countries still face major gaps in diagnostic capacity, limited access to antifungals, and shortages of trained medical personnel^5–8^.

In 2022, the World Health Organization released the Fungal Priority Pathogens List (FPPL), identifying *Cryptococcus neoformans*, *Candida auris*, *Aspergillus fumigatus*, and others as critical priority group of pathogens requiring urgent action to reduce morbidity and mortality^9^. The FPPL emphasizes not only the microbiological and clinical significance of these fungi but also the structural drivers of poor outcomes, including insufficient diagnostic capacity, inequitable access to antifungal therapy, and weak surveillance systems. This framework strongly aligns with challenges historically observed in LAC, where several WHO high-priority fungi are endemic or increasingly detected, yet public health responses remain fragmented and under-resourced. Thus, the WHO FPPL provides a critical lens through which to interpret current gaps in laboratory readiness and therapeutic access across the region.

A 2019 survey in LAC^2^, which evaluated IFD diagnosis and treatment, showed that only 9% of centers met basic mycology standards. It also revealed serious deficiencies in laboratory infrastructure, limited access to antifungals, and a shortage of trained professionals. This demonstrated the need to strengthen diagnostic tools and ensure access to safe and effective antifungals throughout the region^8^. Since then, advancements have been uneven across LAC. It is difficult to plan effectively and determine where resources are most needed across the region. While the World Health Organization (WHO) has called for minimum standards in diagnosing and treating IFD^9,10^, efforts to implement these guidelines across LAC have been inconsistent. At the same time, rising antifungal resistance and the urgent need for molecular diagnostics further highlight the necessity of up-to-date, region-specific information, precisely the challenge underscored in the WHO FPPL.

This study aims to pinpoint remaining gaps, highlight disparities between countries, and assess how the region’s current mycology capacity aligns with global priorities identified in the WHO FPPL. By doing so, we aim to generate evidence that can guide practical actions toward strengthening regional preparedness for high-risk fungi of major public health importance.

## Results

**Fig. 1:**
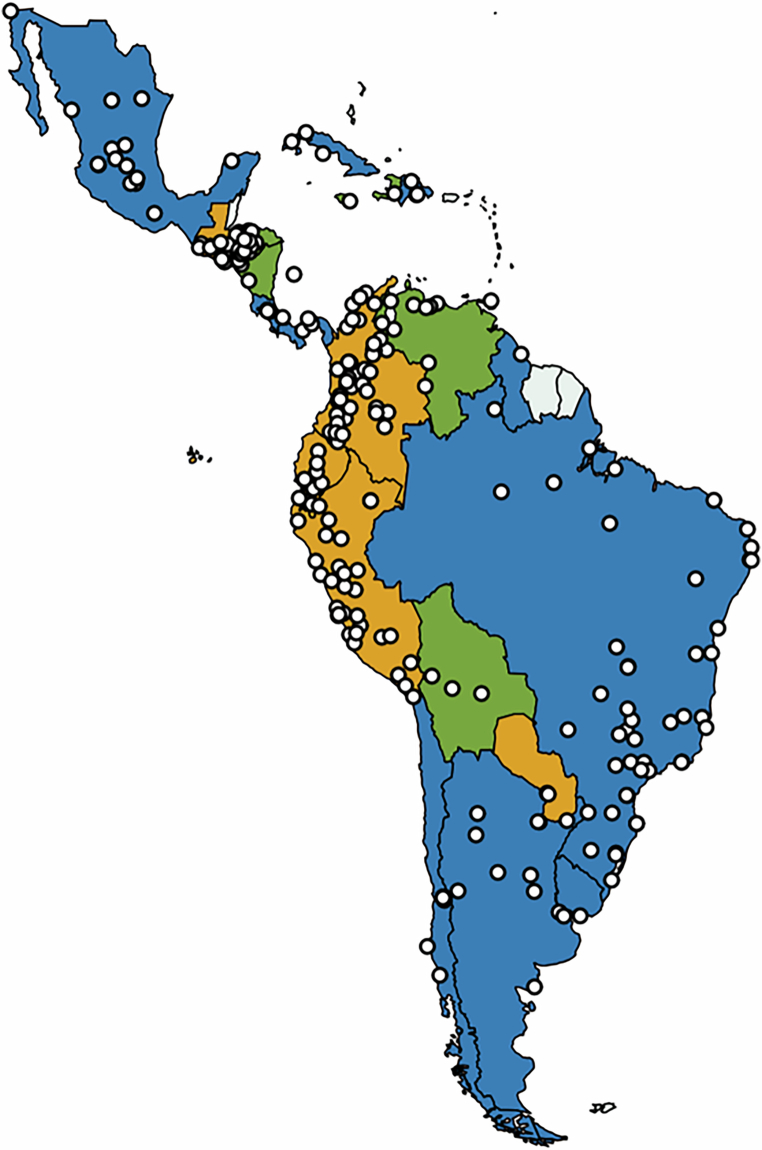
Geographical site distribution of participating countries and institutions from Latin America and the Caribbean. In cases where there is more than one participating center in the same location, a single point is pictured in white. Number of sites per country/territory: Gray Countries/territories with no participating institutions colored in gray: Anguilla, Antigua and Barbuda, Aruba, Bahamas, Barbados, Belize, Bermuda, Bonaire, British Virgin Islands, Cayman Islands, Curaçao, Dominica, Falkland Islands, French Guyana, Grenada, Montserrat, Puerto Rico, Saba, Saint Kitts and Nevis, Saint Lucia, Saint Martin, Saint Vincent and the Grenadines, Saint-Barthélemy, Sint Eustatius, Sint Maarten, Suriname, Turks and Caicos Islands, and United States Virgin Islands (*n* = 0, each). Green Countries/territories with nominal gross domestic product (GDP) < US$ 5000 are colored in green: Honduras (*n* = 80), Venezuela (*n* = 12), Bolivia (*n* = 6), and Haiti, Jamaica, and Nicaragua (*n* = 1, each). Yellow Countries/territories with nominal GDP < US$ 5000–10,000 are colored in yellow: Colombia (*n* = 205), Peru (*n* = 80), Ecuador (*n* = 13), Guatemala (*n* = 11), Paraguay (*n* = 5), and El Salvador (*n* = 2). Blue Countries/territories with nominal GDP > US$ 10,000 are colored in blue: Brazil (*n* = 88), Mexico (*n* = 42), Argentina (*n* = 30), Chile (*n* = 10), Cuba (*n* = 10), Dominican Republic (*n* = 7), Panama (*n* = 6), Costa Rica (*n* = 3), and Guyana, Trinidad and Tobago, and Uruguay (*n* = 1 each).

A total of 619 participants from LAC completed the survey. Most of the responses came from Colombia (*n* = 205/619; 33.1%), followed by Brazil (*n* = 88/619; 14.2%), Honduras (*n* = 80/619; 12.9%), Peru (*n* = 80/619; 12.9%), and Mexico (*n* = 42/619; 6.8%) (Supplementary Table 1). The geographical distribution of the participating centers in this study is shown in Fig. 1.

Of the 619 participating institutions, 617 responded to the question regarding self-perceived incidence of IFD, 30.3% (*n* = 187/617) reported it as very low, 24.3% (*n* = 150/617) as low, and 32.4% (*n* = 200/617) as moderate. When asked which fungi posed the highest risk in their settings, *Candida* spp. were most frequently identified (*n* = 572/619; 92.4%), followed by *Aspergillus* spp. (*n* = 334/619; 54.0%) and *Cryptococcus* spp. (*n* = 314/619; 50.7%). Less commonly mentioned were *Histoplasma* spp. (*n* = 240/619; 38.8%), Mucorales (*n* = 110/619; 17.8%), and *Fusarium* spp. (*n* = 76/619; 12.3%) (Table 1).

**Table 1:** Perceived incidence and priority fungal pathogens identified as high-risk in Latin America and the Caribbean

	Overall		>US$ 5000		>US$ 5000–10,000		>US$ 10,000		*p* value
	*n* = 619		*n* = 101		*n* = 318		*n* = 200		
	*n*	*%*	*n*	*%*	*n*	*%*	*n*	*%*	
**IFD incidence self-perception**									<0.001
Very low	187/617	30.3	46/100	46.0	114/318	35.8	27/199	13.6	
Low	150/617	24.3	21/100	21.0	73/318	23.0	56/199	28.1	
Moderate	200/617	32.4	20/100	20.0	99/318	31.1	81/199	40.7	
High	56/617	9.1	8/100	8.0	24/318	7.5	24/199	12.1	
Very high	24/617	3.9	5/100	5.0	8/318	2.5	11/199	5.5	
**Fungi perceived as of highest risk**									
* Aspergillus* spp.	334/619	54.0	39/101	38.6	158/318	49.7	137/200	68.5	<0.001
* Candida* spp.	572/619	92.4	95/101	94.1	295/318	92.8	182/200	91.0	0.657
* Cryptococcus* spp.	314/619	50.7	27/101	26.7	166/318	52.2	121/200	60.5	<0.001
* Fusarium* spp.	76/619	12.3	13/101	12.9	28/318	8.8	35/200	17.5	0.014
* Histoplasma spp*.	240/619	38.8	25/101	24.8	121/318	38.1	94/200	47.0	<0.001
* Lomentospora*/*Scedosporium* spp.	10/591	1.7	5/100	5.0	2/312	0.6	3/179	1.7	0.015
Mucorales	110/619	17.8	18/101	17.8	42/318	13.2	50/200	25.0	0.003
Phaeohyphomycetes	24/591	4.1	6/100	6.0	9/312	2.9	9/179	5.0	0.263

*IFD* invasive fungal diseases, *n* number (sample size), *p* probability, *spp.* species, *US$* United States dollar.

**Fig. 2:**
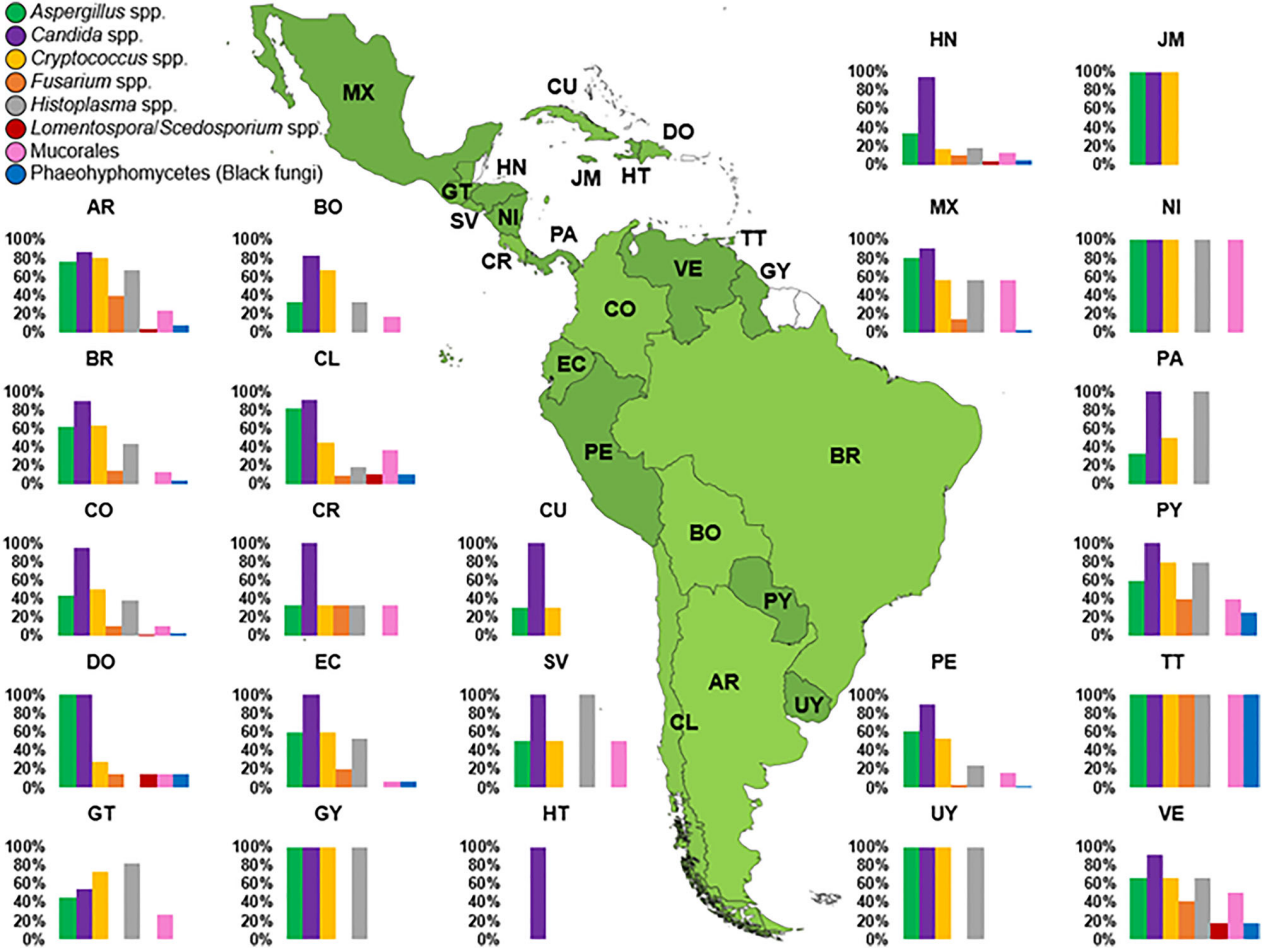
Ranking of fungi perceived as those of greatest concern in Latin America and the Caribbean. The figure provides a ranking of fungal pathogens identified as high-risk across various countries. Country abbreviations include AR (Argentina), BO (Bolivia), BR (Brazil), CL (Chile), CO (Colombia), CR (Costa Rica), CU (Cuba), DO (Dominican Republic), EC (Ecuador), GT (Guatemala), GY (Guyana), HT (Haiti), HN (Honduras), JM (Jamaica), MX (Mexico), NI (Nicaragua), PA (Panama), PY (Paraguay), PE (Peru), SV (El Salvador), TT (Trinidad and Tobago), UY (Uruguay), and VE (Venezuela).

**Fig. 3:**
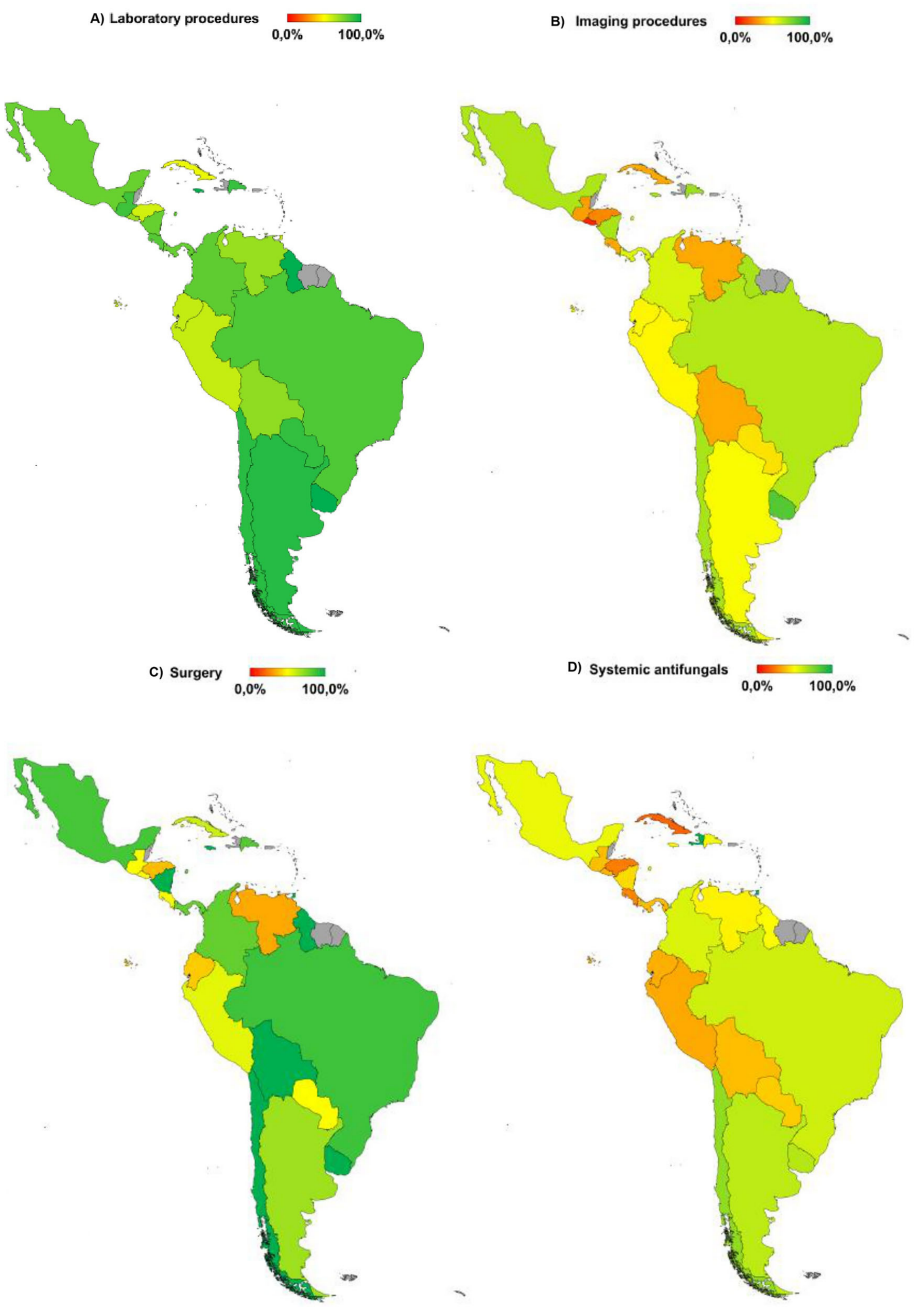
Heat maps of the pooled access to laboratory procedures, imaging procedures, surgery, and antifungal treatment per country. The figure illustrates regional access to critical medical resources across four panels: **A** laboratory procedures; **B** Imaging procedures; **C** Surgery; **D** systemic antifungals. The color scale represents the percentage of access, ranging from 0.0% to 100.0%.

Access to diagnostic tools among the 497 participating centers is summarized in Table 2. Microscopy was widely available (*n* = 475/497; 95.6%), but fluorescence microscopy was limited (*n* = 59/497; 11.9%). Direct microscopic examination included KOH wet mount preparation (*n* = 405/464; 87.3%), India/China ink (*n* = 408/493; 82.8%), Giemsa (*n* = 255/461; 55.3%), and silver stain (*n* = 134/451; 29.7%). Fungal cultures were available in 89.4% (*n* = 428/479); lack of culture access was reported in Bolivia, Colombia, Ecuador, Guatemala, Honduras, Mexico, Peru, and Venezuela. Common media included Sabouraud agar (75.5%), Sabouraud with chloramphenicol (48.3%), and Sabouraud with gentamicin (29.3%). Species identification was possible in 76.2% of centers, mainly via automated systems (51.5%) and biochemical tests (49.8%), while MALDI–TOF–MS was available in 23.8%. Antifungal susceptibility testing was performed in 67.7%, mostly using automated methods (49.3%) (Figs. 2 and 3).

**Table 2:** Access to diagnostic tools for invasive fungal infections in Latin America and the Caribbean

	Overall		>US$ 5000		>US$ 5000–10,000		>US$ 10,000		*p* value
	*n* = 619		*n* = 101		*n* = 318		*n* = 200		
	*n*	*%*	*n*	*%*	*n*	*%*	*n*	*%*	
**Microscopy**	475/497	95.6	77/84	91.7	213/225	94.7	185/188	98.4	0.022
Stains									
Calcofluor white	59/236	25.0	3/22	13.6	17/75	22.7	39/139	28.1	0.330
Giemsa	255/461	55.3	31/82	37.8	113/220	51.4	111/159	69.8	<0.001
China/India ink	408/493	82.8	44/83	53.0	183/222	82.4	181/188	96.3	<0.001
KOH	405/464	87.3	71/79	89.9	192/219	87.7	142/166	85.5	0.636
Silver	134/451	29.7	6/80	7.5	43/205	21.0	85/166	51.2	<0.001
Access to fluorescence	59/497	11.9	1/84	1.2	19/225	8.4	39/188	20.7	<0.001
If suspicion of…									
Cryptococcosis: Direct exam of body fluids	417/440	94.8	56/60	93.3	181/192	94.3	180/188	95.7	0.637
Pneumocystosis: Silver staining	117/497	23.5	8/84	9.5	38/225	16.9	71/188	37.8	<0.001
Mucormycosis: Direct microscopy	68/483	14.1	11/84	13.1	14/222	6.3	43/177	24.3	<0.001
**Culture**	428/479	89.4	63/79	79.7	181/215	84.2	184/185	99.5	<0.001
Culture media									
Agar Niger	65/232	28.0	4/27	14.8	14/71	19.7	47/134	35.1	0.020
Chromogen	211/393	53.7	25/76	32.9	88/184	47.8	98/133	73.7	<0.001
Lactrimel	65/380	17.1	11/74	14.9	12/176	6.8	42/130	32.3	<0.001
Potato agar	148/398	37.2	12/75	16.0	50/182	27.5	86/141	61.0	<0.001
Saboraud agar	332/440	75.5	49/77	63.6	135/202	66.8	148/161	91.9	<0.001
Saboraud agar + Chloramphenicol	190/393	48.3	20/74	27.0	80/179	44.7	90/140	64.3	<0.001
Saboraud agar + Gentamicine	114/389	29.3	13/74	17.6	39/176	22.2	62/139	44.6	<0.001
Selective agar (Chloramphenicol + Cycloheximide)	120/385	31.2	15/74	20.3	36/174	20.7	69/137	50.4	<0.001
Available tests for species identification	353/463	76.2	34/78	43.6	147/208	70.7	172/177	97.2	<0.001
Automated identification	234/454	51.5	15/78	19.2	84/203	41.4	135/173	78.0	<0.001
Biochemical tests	220/442	49.8	21/78	26.9	77/198	38.9	122/166	73.5	<0.001
DNA sequencing	80/445	18.0	2/77	2.6	32/204	15.7	46/164	28.0	<0.001
MALDI–TOF–MS	107/449	23.8	3/76	3.9	27/203	13.3	77/170	45.3	<0.001
Mounting medium	100/401	24.9	15/73	20.5	50/191	26.2	35/137	25.5	0.652
Available antifungal susceptibility test technologies	323/477	67.7	21/79	26.6	143/214	66.8	159/184	86.4	
Broth microdilution, using CLSI standards	122/417	29.3	9/76	11.8	39/194	20.1	74/147	50.3	<0.001
Broth microdilution, using EUCAST standards	56/410	13.7	2/75	2.7	9/188	4.8	45/147	30.6	<0.001
Gradient diffusion test	98/408	24.0	8/76	10.5	26/187	13.9	64/145	44.1	<0.001
Automated identification	214/434	49.3	13/78	16.7	96/194	49.5	105/162	64.8	<0.001
**Serology**	213/307	69.4	16/28	57.1	59/105	56.2	138/174	79.3	<0.001
*Aspergillus* spp.	184/302	60.9	11/26	42.3	49/103	47.6	124/173	71.7	<0.001
Onsite	100/302	33.1	6/26	23.1	22/103	21.4	72/173	41.6	
Outsourced	84/302	27.8	5/26	19.2	27/103	26.2	52/173	30.1	
*Candida* spp.	93/293	31.7	8/26	30.8	29/100	29.0	56/167	33.5	0.782
Onsite	42/293	14.3	4/26	15.4	9/100	9.0	29/167	17.4	
Outsourced	51/293	17.4	4/26	15.4	20/100	20.0	27/167	16.2	
*Histoplasma* spp.	188/304	61.8	15/28	53.6	54/103	52.4	119/173	68.8	0.017
Onsite	95/304	31.3	8/28	28.6	22/103	21.4	65/173	37.6	
Outsourced	93/304	30.6	7/28	25.0	32/103	31.1	54/173	31.2	
*Paracoccidioides* spp.	140/293	47.8	12/27	44.4	39/100	39.0	89/166	53.6	0.065
Onsite	62/293	21.2	6/27	22.2	11/100	11.0	45/166	27.1	
Outsourced	78/293	26.6	6/27	22.2	28/100	28.0	44/166	26.5	
**Antigen detection**	318/413	77.0	29/35	82.9	133/205	64.9	156/173	90.2	<0.001
*Aspergillus* spp. GM	253/404	62.6	17/30	56.7	105/204	51.5	131/170	77.1	<0.001
* Aspergillus* spp. GM (ELISA)	184/328	56.1	9/29	31.0	83/142	58.5	92/157	58.6	0.019
Onsite	82/328	25.0	2/29	6.9	29/142	20.4	51/157	32.5	
Outsourced	102/328	31.1	7/29	24.1	54/142	38.0	41/157	26.1	
* Aspergillus* spp. GM (LFA)	151/384	39.3	12/29	41.4	70/200	35.0	69/155	44.5	0.183
Onsite	63/384	16.4	6/29	20.7	21/200	10.5	36/155	23.2	
Outsourced	88/384	22.9	6/29	20.7	49/200	24.5	33/155	21.3	
* Aspergillus* spp. GM (LFD)	123/291	42.3	9/26	34.6	32/101	31.7	82/164	50.0	0.009
Onsite	62/291	21.3	5/26	19.2	14/101	13.9	43/164	26.2	
Outsourced	61/291	21.0	4/26	15.4	18/101	17.8	39/164	23.8	
*Candida* spp.	86/388	22.2	9/27	33.3	49/202	24.3	28/159	17.6	0.103
Onsite	17/388	4.4	4/27	14.8	7/202	3.5	6/159	3.8	
Outsourced	69/388	17.8	5/27	18.5	42/202	20.8	22/159	13.8	
*Cryptococcus* spp. GM	286/406	70.4	19/32	59.4	126/203	62.1	141/171	82.5	<0.001
* Cryptococcus* spp. GM (LAT)	218/391	55.8	12/29	41.4	90/193	46.6	116/169	68.6	<0.001
Onsite	129/391	33.0	8/29	27.6	42/193	21.8	79/169	46.7	
Outsourced	89/391	22.8	4/29	13.8	48/193	24.9	37/169	21.9	
* Cryptococcus* spp. GM (LFA)	199/395	50.4	14/31	45.2	93/201	46.3	92/163	56.4	0.135
Onsite	130/395	32.9	7/31	22.6	53/201	26.4	70/163	42.9	
Outsourced	69/395	17.5	7/31	22.6	40/201	19.9	22/163	13.5	
*Histoplasma* spp.	206/403	51.1	16/31	51.6	94/203	46.3	96/169	56.8	0.133
Onsite	73/403	18.1	7/31	22.6	23/203	11.3	43/169	25.4	
Outsourced	133/403	33.0	9/31	29.0	71/203	35.0	53/169	31.4	
β-D-glucan	62/391	15.9	6/28	21.4	13/200	6.5	43/163	26.4	<0.001
Onsite	10/391	2.6	2/28	7.1	1/200	0.5	7/163	4.3	
Outsourced	52/391	13.3	4/28	14.3	12/200	6.0	36/163	22.1	
**Molecular tests**	166/388	42.8	6/81	7.4	79/141	56.0	81/166	48.8	<0.001
*Aspergillus* spp.	118/316	37.3	4/24	16.7	58/131	44.3	56/161	34.8	<0.001
Onsite	39/316	12.3	2/24	8.3	11/131	8.4	26/161	16.1	
Outsourced	79/316	25.0	2/24	8.3	47/131	35.9	30/161	18.6	
*Candida* spp.	109/315	34.6	5/25	20.0	59/131	45.0	45/159	28.3	<0.001
Onsite	41/315	13.0	2/25	8.0	17/131	13.0	22/159	13.8	
Outsourced	68/315	21.6	3/25	12.0	42/131	32.1	23/159	14.5	
*Pneumocystis jirovecii*	135/327	41.3	5/25	20.0	67/137	48.9	63/165	38.2	<0.001
Onsite	45/327	13.8	2/25	8.0	13/137	9.5	30/165	18.2	
Outsourced	90/327	27.5	3/25	12.0	54/137	39.4	33/165	20.0	
Mucorales	84/292	28.8	2/24	8.3	48/123	39.0	34/145	23.4	<0.001
Onsite	15/292	5.1	0/24	0.0	4/123	3.3	11/145	7.6	
Outsourced	69/292	23.6	2/24	8.3	44/123	35.8	23/145	15.9	
**Imaging procedures**									
CT	283/372	76.1	14/40	35.0	137/179	76.5	132/153	86.3	<0.001
PET CT	72/372	19.4	2/40	5.0	30/179	16.8	40/153	26.1	0.004
MRI	204/372	54.8	11/40	27.5	85/179	47.5	108/153	70.6	<0.001
PET MRI	24/372	6.5	1/40	2.5	14/179	7.8	9/153	5.9	0.513
Ultrasound	316/372	84.9	24/40	60.0	154/179	86.0	138/153	90.2	<0.001
X-ray	299/372	80.4	23/40	57.5	142/179	79.3	134/153	87.6	<0.001

*CLSI* Clinical and Laboratory Standards Institute, *CT* computed tomography, *DNA* deoxyribonucleic acid, *ELISA* enzyme-linked immunosorbent assay, *EUCAST* European Committee on Antimicrobial Susceptibility Testing, *GM* galactomannan, *KOH* potassium hydroxide, *LAT* latex agglutination test, *LFA* lateral flow assay, *LFD* lateral flow device, *MALDI–TOF–MS* matrix-assisted laser desorption/ionization time-of-flight mass spectrometry, *MRI* magnetic resonance imaging, *n* number (sample size), *PET* positron emission tomography, *p* probability, *spp.* species, *US$* United States dollar.

Serological testing was available in 69.4% of centers, with *Aspergillus* spp. serology most common (60.9%), performed in-house in 100 centers and externally in 84. *Histoplasma* spp. serology was available in 61.8%, and *Paracoccidioides* spp. in 47.8%. Antigen detection was reported in 77.0% of centers. For *Aspergillus* spp., 62.6% used galactomannan assays, mainly ELISA (56.1%), followed by lateral flow assay (39.3%) and lateral flow device (42.3%). *Cryptococcus* spp. antigen was available in 70.4%, primarily via latex agglutination (55.8%). *Candida* spp. antimannan (22.2%) and *Histoplasma* spp. antigen (51.1%) were less common, and β-D-glucan testing was available in 15.9%. Molecular diagnostics were reported in 42.8%, mainly for *Pneumocystis jirovecii* (35.2%) and *Aspergillus* spp. (31.7%), often performed externally (Table 2).

Imaging techniques were broadly accessible among 372 centers. Ultrasound (*n* = 316/372; 84.9%), X-ray (*n* = 299/372; 80.4%), computed tomography (CT) scan (*n* = 283/372; 76.1%), and magnetic resonance imaging (MRI) (*n* = 204/372; 54.8%) were the most common (Table 2).

Surgical procedures for diagnostic or therapeutic purposes were performed in 72.8% of centers (*n* = 228/313). Among 436 respondents, fluconazole was nearly universally available (96.6%), itraconazole (66.8%) and voriconazole (64.9%) were moderately accessible, while newer triazoles, isavuconazole (38.9%) and posaconazole (29.2%), were less common. Amphotericin B (any formulation) was available in 88.1%, mainly as deoxycholate (75.8%) and liposomal (59.9%). Echinocandins were present in 68.8%, with caspofungin (48.7%) and anidulafungin (42.4%) most common. Other antifungals included flucytosine (21.5%) and terbinafine (32.2%). Therapeutic drug monitoring (TDM) was reported in 35.7% of centers, mostly for voriconazole (27.8%), itraconazole (22.8%), and posaconazole (12.4%). TDM for triazoles was typically in-house, whereas other antifungals were generally monitored externally (Table 3).

**Table 3:** Access to antifungal treatments and therapeutic tools in Latin America and the Caribbean

	Overall		>US$ 5000		>US$ 5000–10,000		>US$ 10,000		*p* value
	*n* = 619		*n* = 101		*n* = 318		*n* = 200		
	*n*	*%*	*n*	*%*	*n*	*%*	*n*	*%*	
**Surgery**	228/313	72.8	12/28	42.9	99/144	68.8	117/141	83.0	<0.001
**Systemic antifungals**									
Amphotericin B	378/429	88.1	26/41	63.4	165/195	84.6	187/193	96.9	<0.001
eoxycholate	317/418	75.8	22/39	56.4	137/193	71.0	158/186	84.9	<0.001
Lipid-based formulations	271/425	63.8	13/41	31.7	108/193	56.0	150/191	78.5	<0.001
Lipidic complex	119/405	29.4	6/39	15.4	47/189	24.9	66/177	37.3	0.005
Liposomal	251/419	59.9	11/40	27.5	99/191	51.8	141/188	75.0	<0.001
Echinocandins	295/429	68.8	19/40	47.5	124/196	63.3	152/193	78.8	<0.001
Anidulafungin	175/413	42.4	10/38	26.3	67/192	34.9	98/183	53.6	<0.001
Caspofungin	202/415	48.7	11/38	28.9	110/194	56.7	81/183	44.3	0.002
Micafungin	91/413	22.0	4/38	10.5	22/192	11.5	65/183	35.5	<0.001
Triazoles	424/436	97.2	39/43	90.7	193/198	97.5	192/195	98.5	0.035
Fluconazole	421/436	96.6	38/43	88.4	191/198	96.5	192/195	98.5	0.009
Mold-active triazoles	375/431	87.0	30/41	73.2	168/197	85.3	177/193	91.7	
Itraconazole	284/425	66.8	19/40	47.5	122/196	62.2	143/189	75.7	<0.001
Isavuconazole	159/409	38.9	11/38	28.9	78/189	41.3	70/182	38.5	0.367
Posaconazole	119/408	29.2	1/38	2.6	70/192	36.5	48/178	27.0	<0.001
Voriconazole	274/422	64.9	19/40	47.5	110/193	57.0	145/189	76.7	<0.001
Flucytosine	88/409	21.5	1/38	2.6	45/190	23.7	42/181	23.2	0.004
Terbinafine	129/401	32.2	18/39	46.2	50/185	27.0	61/177	34.5	0.045
**Therapeutic drug monitoring**	121/363	33.3	9/34	26.5	46/170	27.1	66/159	41.5	0.030
Flucytosine	15/333	4.5	0/31	0.0	7/156	4.5	8/146	5.5	0.537
Onsite	9/333	2.7	0/31	0.0	1/156	0.6	8/146	5.5	
Outsourced	6/333	1.8	0/31	0.0	6/156	3.8	0/146	0.0	
Isavuconazole	22/303	7.3	4/28	14.3	11/148	7.4	7/127	5.5	0.244
Onsite	8/303	2.6	1/28	3.6	4/148	2.7	3/127	2.4	
Outsourced	14/303	4.6	3/28	10.7	7/148	4.7	4/127	3.1	
Itraconazole	74/325	22.8	5/27	18.5	33/151	21.9	36/147	24.5	0.779
Onsite	44/325	13.5	5/27	18.5	14/151	9.3	25/147	17.0	
Outsourced	30/325	9.2	0/27	0.0	19/151	12.6	11/147	7.5	
Posaconazole	36/318	11.3	0/27	0.0	19/151	12.6	17/140	12.1	0.130
Onsite	14/318	4.4	0/27	0.0	6/151	4.0	8/140	5.7	
Outsourced	22/318	6.9	0/27	0.0	13/151	8.6	9/140	6.4	
Voriconazole	94/338	27.8	4/29	13.8	34/158	21.5	56/151	37.1	0.002
Onsite	57/338	16.9	4/29	13.8	12/158	7.6	41/151	27.2	
Outsourced	37/338	10.9	0/29	0.0	22/158	13.9	15/151	9.9	

*n* number (sample size), *p* probability, *US$* United States dollar.

### Comparison by nominal GDP

In countries with nominal GDP < US$ 5000, perceived IFD incidence was mostly low or very low, whereas centers in countries >US$ 10,000 reported medium to high incidence (*p* < 0.001) (Table 1). Pathogens such as *Aspergillus* spp. (*p* < 0.001), *Cryptococcus* spp. (*p* < 0.001), *Histoplasma* spp. (*p* < 0.001), and Mucorales (*p* = 0.003) were more commonly identified in higher-GDP settings.

Basic microscopy was widely available, though accessibility varied (*p* = 0.022). Specialized stains (China/India ink, Giemsa, silver, fluorescent microscopy) and fungal cultures—including enriched/selective media and fungus-specific cultures—were significantly more available in higher-GDP countries (all p < 0.001) (Table 2). Species-level identification and advanced techniques (MALDI–TOF–MS, sequencing, automated systems) were also more accessible in GDP > US$ 10,000 centers (all *p* < 0.001), as was antifungal susceptibility testing and standardized methods (EUCAST, CLSI, gradient strips; all *p* < 0.001).

Serological testing, antigen detection (GM assays, cryptococcal antigen), β-D-glucan, and molecular diagnostics were significantly more available in GDP > US$ 10,000 settings (all *p* < 0.001), with molecular tests also higher in GDP > US$ 5000 countries. Imaging (CT, MRI, ultrasound) and surgical interventions were likewise more accessible in higher-GDP settings (*p* < 0.001).

Access to antifungals increased with GDP: amphotericin B (all formulations), echinocandins (anidulafungin *p* < 0.001, caspofungin *p* = 0.002, micafungin *p* < 0.001), triazoles (voriconazole, itraconazole *p* < 0.001; general triazoles *p* = 0.035), posaconazole (*p* < 0.001), and flucytosine (*p* = 0.004). Fluconazole access was near-universal (*p* = 0.009), while terbinafine was more common in GDP < US$ 5000 (*p* = 0.045). TDM was reported more frequently in GDP > US$ 10,000, especially for voriconazole (*p* = 0.030 and *p* = 0.002) (Table 3).

### Comparative assessment of fungal diagnostic capacity and antifungal access in centers with and without transplant performance

Perceived IFD incidence differed notably between centers. Non-HSCT/SOT facilities mostly reported very low to mild caseloads, whereas transplant centers ranged from mild to very high (*p* < 0.001). *Aspergillus* spp., *Fusarium* spp., and Mucorales were more often considered high-risk in HSCT/SOT settings (all *p* < 0.001) (Table 4).

**Table 4:** Perceived incidence, high-risk fungal pathogens, and access to diagnostic tools and antifungal treatments in Latin America and the Caribbean, per type of patient attended in the participating institutions

	HSCT/SOT		Non-HSCT/SOT			HIV		Non-HIV		
	*n* = 162		*n* = 198		*p* value	*n* = 408		*n* = 24		*p* value
	*n*	*%*	*n*	*%*		*n*	*%*	*n*	*%*	
**IFI incidence self-perception**					<0.001					0.406
Very low	9/162	5.6	57/197	28.9		82/407	20.1	6/24	25.0	
Low	39/162	24.1	52/197	26.4		101/407	24.8	9/24	37.5	
Mild	82/162	50.6	60/197	30.5		158/407	38.8	6/24	25.0	
High	22/162	13.6	18/197	9.1		44/407	10.8	3/24	12.5	
Very high	10/162	6.2	10/197	5.1		22/407	5.4	0/24	0.0	
**Fungi perceived as of highest risk**										
*Aspergillus* spp.	136/162	84.0	96/198	48.5	<0.001	255/408	62.5	12/24	50.0	0.280
*Candida* spp.	151/162	93.2	177/198	89.4	0.264	375/408	91.9	23/24	95.8	0.710
*Cryptococcus* spp.	109/162	67.3	113/198	57.1	0.051	253/408	62.0	7/24	29.2	0.002
*Fusarium* spp.	36/162	22.2	15/198	7.6	<0.001	51/408	12.5	4/24	16.7	0.529
*Histoplasma spp*.	86/162	53.1	91/198	46.0	0.204	199/408	48.8	7/24	29.2	0.091
*Lomentospora*/*Scedosporium* spp.	1/140	0.7	5/194	2.6	0.407	8/382	2.1	0/23	0.0	1.000
Mucorales	52/162	32.1	32/198	16.2	<0.001	89/408	21.8	6/24	25.0	0.800
Phaeohyphomycetes	10/140	7.1	6/194	3.1	0.118	17/382	4.5	1/23	4.3	1.000
**Microscopy**	134/137	97.8	180/184	97.8	1.000	299/306	97.7	22/23	95.7	0.444
Stains										
Calcofluor white	35/106	33.0	23/119	19.3	0.022	53/212	25.0	4/19	21.1	1.000
Giemsa	94/125	75.2	120/161	74.5	1.000	207/273	75.8	13/21	61.9	0.191
China/India unk	131/135	97.0	171/182	94.0	0.286	290/302	96.0	19/23	82.6	0.020
KOH	115/128	89.8	130/160	81.3	0.047	233/274	85.0	19/22	86.4	1.000
Silver	72/124	58.1	52/152	34.2	<0.001	121/264	45.8	5/20	25.0	0.101
Access to fluorescence	30/137	21.9	21/184	11.4	0.013	49/306	16.0	2/23	8.7	0.550
If suspicion of…										
Cryptococcosis: Direct exam of body fluids	132/137	96.4	167/184	90.8	0.072	289/306	94.4	17/23	73.9	0.003
Pneumocystis: Silver stain	59/137	43.1	42/184	22.8	<0.001	97/306	31.7	5/23	21.7	0.361
Mucormycosis: Direct microscopy	33/126	26.2	27/182	14.8	0.019	56/293	19.1	3/22	13.6	0.777
**Culture**	132/133	99.2	166/177	93.8	0.011	284/295	96.3	22/24	91.7	0.255
Culture media										
Agar Niger	38/97	39.2	26/123	21.1	0.004	61/210	29.0	4/17	23.5	0.784
Chromogen	77/99	77.8	66/127	52.0	<0.001	142/214	66.4	8/19	42.1	0.045
Lactrimel	31/95	32.6	26/120	21.7	0.087	55/202	27.2	6/20	30.0	0.796
Potato agar	68/107	63.6	46/125	36.8	<0.001	112/219	51.1	7/20	35.0	0.242
Saboraud agar	118/126	93.7	123/146	84.2	0.021	229/259	88.4	19/21	90.5	1.000
Saboraud agar + Chloramphenicol	67/104	64.4	64/123	52.0	0.079	126/215	58.6	10/19	52.6	0.635
Saboraud agar + Gentamicine	44/103	42.7	46/120	38.3	0.584	86/211	40.8	7/19	36.8	0.811
Selective agar (Chloramphenicol + Cycloheximide)	51/100	51.0	43/118	36.4	0.039	95/206	46.1	7/19	36.8	0.480
Available tests for species identification	127/130	97.7	140/165	84.8	<0.001	256/282	90.8	18/21	85.7	0.437
Automated identification	106/127	83.5	98/159	61.6	<0.001	199/273	72.9	12/21	57.1	0.135
Biochemical tests	89/124	71.8	103/150	68.7	0.598	184/264	69.7	12/18	66.7	0.794
DNA sequencing	33/122	27.0	24/155	15.5	0.024	50/264	18.9	7/21	33.3	0.151
MALDI–TOF–MS	65/126	51.6	33/155	21.3	<0.001	94/269	34.9	5/20	25.0	0.468
Mounting medium	24/97	24.7	16/139	11.5	0.013	41/224	18.3	2/19	10.5	0.540
Available antifungal susceptibility test technologies	122/133	91.7	120/175	68.6		237/294	80.6	13/23	56.5	
Broth microdilution. using CLSI standards	53/109	48.6	56/141	39.7	0.198	106/236	44.9	4/21	19.0	0.022
Broth microdilution. using EUCAST standards	31/109	28.4	22/135	16.3	0.028	54/230	23.5	0/21	0.0	0.010
Gradient diffusion test	53/110	48.2	32/131	24.4	<0.001	84/227	37.0	4/21	19.0	0.151
Automated identification	79/119	66.4	73/149	49.0	0.005	150/254	59.1	7/21	33.3	0.037
**Serology**	101/128	78.9	101/166	60.8	<0.001	196/279	70.3	12/22	54.5	0.151
*Aspergillus* spp.	90/126	71.4	85/163	52.1	0.001	173/275	62.9	7/22	31.8	0.006
Onsite	52/126	41.3	39/163	23.9		93/275	33.8	3/22	13.6	
Outsourced	38/126	30.2	46/163	28.2		80/275	29.1	4/22	18.2	
*Candida* spp.	39/123	31.7	51/159	32.1	1.000	89/268	33.2	2/22	9.1	0.017
Onsite	15/123	12.2	24/159	15.1		40/268	14.9	0/22	0.0	
Outsourced	24/123	19.5	27/159	17.0		49/268	18.3	2/22	9.1	
*Histoplasma spp*.	84/127	66.1	93/164	56.7	0.116	172/276	62.3	11/22	50.0	0.264
Onsite	41/127	32.3	45/164	27.4		86/276	31.2	5/22	22.7	
Outsourced	43/127	33.9	48/164	29.3		86/276	31.2	6/22	27.3	
*Paracoccidioides* spp.	64/120	53.3	69/160	43.1	0.093	126/265	47.5	9/22	40.9	0.659
Onsite	32/120	26.7	23/160	14.4		54/265	20.4	4/22	18.2	
Outsourced	32/120	26.7	46/160	28.8		72/265	27.2	5/22	22.7	
**Antigen detection**	121/129	93.8	121/163	74.2	<0.001	235/277	84.8	14/22	63.6	0.017
*Aspergillus* spp. GM	109/128	85.2	81/157	51.6	<0.001	187/271	69.0	8/22	36.4	0.004
* Aspergillus* spp. GM (ELISA)	75/121	62.0	57/148	38.5	<0.001	130/256	50.8	5/21	23.8	0.022
Onsite	50/121	41.3	15/148	10.1		65/256	25.4	2/21	9.5	
Outsourced	25/121	20.7	42/148	28.4		65/256	25.4	3/21	14.3	
* Aspergillus* spp. GM (LFA)	56/119	47.1	50/147	34.0	0.033	103/253	40.7	6/21	28.6	0.356
Onsite	36/119	30.3	15/147	10.2		51/253	20.2	2/21	9.5	
Outsourced	20/119	16.8	35/147	23.8		52/253	20.6	4/21	19.0	
* Aspergillus* spp. GM (LFD)	62/124	50.0	58/154	37.7	0.051	118/265	44.5	5/21	23.8	0.071
* Onsite*	36/124	29.0	23/154	14.9		60/265	22.6	2/21	9.5	
* Outsourced*	26/124	21.0	35/154	22.7		58/265	21.9	3/21	14.3	
*Candida* spp.	20/121	16.5	33/151	21.9	0.285	53/258	20.5	2/22	9.1	0.268
Onsite	2/121	1.7	9/151	6.0		12/258	4.7	0/22	0.0	
Outsourced	18/121	14.9	24/151	15.9		41/258	15.9	2/22	9.1	
*Cryptococcus* spp. GM	111/128	86.7	105/159	66.0	<0.001	210/272	77.2	12/22	54.5	0.035
* Cryptococcus* spp. GM (LAT)	85/126	67.5	82/148	55.4	0.047	157/260	60.4	12/21	57.1	0.819
Onsite	62/126	49.2	49/148	33.1		106/260	40.8	6/21	28.6	
Outsourced	23/126	18.3	33/148	22.3		51/260	19.6	6/21	28.6	
* Cryptococcus* spp. GM (LFA)	75/123	61.0	70/153	45.8	0.015	145/262	55.3	6/22	27.3	0.014
Onsite	56/123	45.5	46/153	30.1		101/262	38.5	4/22	18.2	
Outsourced	19/123	15.4	24/153	15.7		44/262	16.8	2/22	9.1	
*Histoplasma spp*.	72/126	57.1	80/159	50.3	0.283	148/270	54.8	7/22	31.8	0.046
Onsite	30/126	23.8	34/159	21.4		62/270	23.0	4/22	18.2	
Outsourced	42/126	33.3	46/159	28.9		86/270	31.9	3/22	13.6	
β-D-glucan	30/125	24.0	30/150	20.0	0.465	58/261	22.2	2/22	9.1	0.115
Onsite	7/125	5.6	2/150	1.3		9/261	3.4	0/22	0.0	
Outsourced	23/125	18.4	28/150	18.7		49/261	18.8	2/22	9.1	
**Molecular tests**	68/125	54.4	54/153	35.3	0.002	113/264	42.8	10/21	47.6	0.820
*Aspergillus* spp.	48/122	39.3	37/150	24.7	0.012	80/258	31.0	6/21	28.6	1.000
Onsite	21/122	17.2	12/150	8.0		31/258	12.0	3/21	14.3	
Outsourced	27/122	22.1	25/150	16.7		49/258	19.0	3/21	14.3	
*Candida* spp.	37/122	30.3	39/149	26.2	0.498	71/257	27.6	6/21	28.6	1.000
Onsite	16/122	13.1	15/149	10.1		27/257	10.5	5/21	23.8	
Outsourced	21/122	17.2	24/149	16.1		44/257	17.1	1/21	4.8	
*Pneumocystis jirovecii*	55/124	44.4	40/153	26.1	0.002	92/263	35.0	4/21	19.0	0.158
Onsite	25/124	20.2	11/153	7.2		36/263	13.7	1/21	4.8	
Outsourced	30/124	24.2	29/153	19.0		56/263	21.3	3/21	14.3	
Mucorales	28/108	25.9	28/145	19.3	0.224	51/239	21.3	5/20	25.0	0.777
Onsite	7/108	6.5	5/145	3.4		10/239	4.2	2/20	10.0	
Outsourced	21/108	19.4	23/145	15.9		41/239	17.2	3/20	15.0	
**Imaging procedures**										
CT	115/131	87.8	110/156	70.5	<0.001	263/332	79.2	11/21	52.4	0.012
PET CT	51/131	38.9	13/156	8.3	<0.001	69/332	20.8	2/21	9.5	0.272
MRI	109/131	83.2	56/156	35.9	<0.001	191/332	57.5	7/21	33.3	0.040
PET MRI	18/131	13.7	4/156	2.6	<0.001	22/332	6.6	1/21	4.8	1.000
Ultrasound	122/131	93.1	127/156	81.4	0.005	289/332	87.0	16/21	76.2	0.183
X-ray	113/131	86.3	122/156	78.2	0.091	276/332	83.1	15/21	71.4	0.231
**Surgery**	103/113	91.2	81/130	37.7	<0.001	209/281	74.4	13/18	72.2	0.787
**Systemic antifungals**										
Amphotericin B	152/155	98.1	165/188	87.8	<0.001	354/391	90.5	16/20	80.0	0.127
Deoxycholate	125/148	84.5	144/185	77.8	0.161	300/381	78.7	11/20	55.0	0.024
Lipid-based formulations	133/153	86.9	91/188	48.4	<0.001	257/388	66.2	9/20	45.0	0.058
Lipidic complex	52/143	36.4	49/182	26.9	0.071	115/369	31.2	3/20	15.0	0.142
Liposomal	124/149	83.2	83/186	44.6	<0.001	240/382	62.8	7/20	35.0	0.017
Echinocandins	139/155	89.7	99/189	52.4	<0.001	279/391	71.4	10/21	47.6	0.027
Anidulafungin	94/150	62.7	54/182	29.7	<0.001	167/375	44.5	5/21	23.8	0.072
Caspofungin	90/148	60.8	61/185	33.0	<0.001	188/377	49.9	8/21	38.1	0.371
Micafungin	46/145	31.7	33/183	18.0	0.004	88/376	23.4	2/20	10.0	0.271
Triazoles	156/157	99.4	184/192	95.8	0.045	388/398	97.5	20/21	95.2	0.436
Fluconazole	156/157	99.4	183/192	95.3	0.026	386/398	97.0	20/21	95.2	0.493
Mold-active triazoles	150/157	95.5	163/190	85.8		351/393	89.3	15/21	71.4	0.025
Itraconazole	113/153	73.9	145/188	77.1	0.527	268/387	69.3	13/21	61.9	0.476
Isavuconazole	81/148	54.7	30/179	16.8	<0.001	147/371	39.6	5/21	23.8	0.173
Posaconazole	69/145	47.6	24/182	13.2	<0.001	115/371	31.0	3/20	15.0	0.209
Voriconazole	139/155	89.7	91/184	49.5	<0.001	262/385	68.1	7/20	35.0	0.006
Flucytosine	34/148	23.0	30/180	16.7	0.163	87/373	23.3	1/20	5.0	0.057
Terbinafine	52/140	37.1	59/178	33.1	0.479	116/363	32.0	5/20	25.0	0.626
**Therapeutic drug monitoring**	55/136	40.4	44/151	29.1	0.050	115/333	34.5	4/18	22.2	0.321
Flucytosine	6/131	4.6	6/137	4.4	1.000	15/307	4.9	0/16	0.0	1.000
Onsite	5/131	3.8	4/137	2.9		9/307	2.9	0/16	0.0	
Outsourced	1/131	0.8	2/137	1.5		6/307	2.0	0/16	0.0	
Isavuconazole	11/108	10.2	5/129	3.9	0.069	21/280	7.5	1/13	7.7	1.000
Onsite	5/108	4.6	3/129	2.3		8/280	2.9	0/13	0.0	
Outsourced	6/108	5.6	2/129	1.6		13/280	4.6	1/13	7.7	
Itraconazole	28/123	22.8	36/140	25.7	0.666	70/300	23.3	3/16	18.8	1.000
Onsite	15/123	12.2	28/140	20.0		42/300	14.0	2/16	12.5	
Outsourced	13/123	10.6	8/140	5.7		28/300	9.3	1/16	6.3	
Posaconazole	22/120	18.3	7/134	5.2	0.001	32/292	11.0	3/15	20.0	0.393
Onsite	10/120	8.3	3/134	2.2		13/292	4.5	1/15	6.7	
Outsourced	12/120	10.0	4/134	3.0		19/292	6.5	2/15	13.3	
Voriconazole	51/129	39.5	28/141	19.9	<0.001	89/312	28.5	3/15	20.0	0.570
Onsite	33/129	25.6	20/141	14.2		55/312	17.6	2/15	13.3	
Outsourced	18/129	14.0	8/141	5.7		34/312	10.9	1/15	6.7	

*CLSI* Clinical and Laboratory Standards Institute, *CT* computed tomography, *DNA* deoxyribonucleic acid, *ELISA* enzyme-linked immunosorbent assay, *EUCAST* European Committee on Antimicrobial Susceptibility Testing, *GM* galactomannan, *HIV* human immunodeficiency virus, *HSCT* hematopoietic stem cell transplantation, *IFD* invasive fungal disease, *KOH* potassium hydroxide, *LAT* latex agglutination test, *LFA* lateral flow assay, *LFD* lateral flow device, *MALDI–TOF–MS* matrix-assisted laser desorption/ionization time-of-flight mass spectrometry, *MRI* magnetic resonance imaging, *n* number (sample size), *PET* positron emission tomography, *p* probability, *SOT* solid organ transplantation, *spp.* species.

Basic microscopy was widely available, but KOH (*p* = 0.047), and specific stains such as calcofluor white (*p* = 0.022), silver (*p* < 0.001), and fluorescence microscopy (*p* = 0.013) were more common in HSCT/SOT centers. Fungal culture specialized media (chromogenic agar, *p* < 0.001), and advanced identification techniques (MALDI–TOF–MS, *p* < 0.001; DNA sequencing, *p* = 0.024) were also more frequent, as was antifungal susceptibility testing (*p* < 0.001).

Serology, especially for Aspergillus spp., antigen detection (galactomannan and cryptococcal assays, both *p* < 0.001), and molecular diagnostics (*p* = 0.020; *Aspergillus* *p* = 0.012, *Pneumocystis jirovecii* *p* = 0.002) were higher in transplant centers. Imaging (CT, PET CT, MRI, PET MRI, all *p* < 0.001; ultrasound *p* = 0.005) and surgical interventions (*p* < 0.001) followed the same trend.

HSCT/SOT centers had broader antifungal availability: liposomal amphotericin B, echinocandins (all *p* < 0.001 except micafungin *p* = 0.004), triazoles (*p* = 0.045), including isavuconazole, posaconazole, voriconazole (all *p* < 0.001), and fluconazole (*p* = 0.026). TDM was more common for posaconazole (*p* = 0.001) and voriconazole (*p* < 0.001) (Table 4).

### Comparative assessment of fungal diagnostic capacity and antifungal access in centers with and without PLWHA

Significant differences were observed between PLWHA-focused and non-HIV centers. PLWHA facilities were more likely to identify *Cryptococcus* spp. as high-risk (*p* < 0.001) and perform direct microscopy for suspected cryptococcosis (*p* < 0.001). Access to *Cryptococcus* diagnostics was higher, including China/India ink staining (*p* = 0.020) and antigen detection, particularly lateral flow assays (*p* = 0.014, *p* = 0.035).

For *Aspergillus* spp., galactomannan detection was more common in PLWHA-serving centers (*p* < 0.001). Antifungal susceptibility testing using CLSI (*p* = 0.022), EUCAST (*p* = 0.010), or automated systems (*p* = 0.037) was also higher. Antigen detection for *Candida* (*p* = 0.017), *Histoplasma* (*p* = 0.046), and overall fungal antigens (*p* = 0.017) followed the same pattern.

Imaging access was greater, with CT (*p* = 0.012) and MRI (*p* = 0.040) more available. Antifungal treatment access was higher for liposomal amphotericin B (*p* = 0.017), deoxycholate amphotericin B (*p* = 0.024), at least one echinocandin (*p* = 0.027), mold-active triazoles (*p* = 0.025), and voriconazole (*p* < 0.001) (Table 4).

## Discussion

This comprehensive LAC survey highlights a heterogeneous landscape in IFD incidence, diagnostic capacity, and therapeutic availability, with disparities driven by national income and patient risk. *Candida* and *Aspergillus* spp. were the most concerning pathogens. Access to advanced microbiological, serological, molecular, and imaging diagnostics favored higher-GDP countries and transplant centers. Similarly, key antifungals—including liposomal amphotericin B, newer triazoles, and echinocandins—and implementation of TDM were substantially greater in high-income settings and among centers managing high-risk populations, such as HSCT/SOT recipients or PLWHA. These patterns mirror the challenges emphasized by the 2022 WHO Fungal Priority Pathogens List (FPPL), which identifies several of the fungi most frequently reported in our survey (including *Histoplasma capsulatum*, *Cryptococcus neoformans*, *Aspergillus fumigatus*, and drug-resistant *Candida* spp.) as requiring urgent public health action due to the combined effects of high mortality, diagnostic complexity, and treatment limitations.

To our knowledge, this survey is the largest, most representative, and most up-to-date snapshot of the diagnostic and therapeutic capacity in LAC. A previous analysis focused much more on Brazilian institutions than on the rest of the region^2^. In this updated analysis, it has become clearer that, even while facing enormous challenges in terms of incidence, impact, and burden of IFD in the region^4^, institutions still have significant limitations regarding their capacity and infrastructure. The WHO FPPL highlights these gaps as critical barriers to reducing mortality from high-risk fungi, underscoring the need for coordinated policy and system-level interventions rather than isolated, center-specific improvements.

The self-perceived incidence of IFD was moderate to very high in about 45% of institutions overall, rising to 70% in institutions that perform transplants. This corroborates the already well-established knowledge that the region faces a major challenge ahead, especially regarding endemic mycoses^11^, although species of *Candida* spp. (92%) and *Aspergillus* spp. (54%) are considered the most concerning fungi by respondents, and in line with surveys from other regions^12–14^, *Cryptococcus* spp. (51%) and *Histoplasma* spp. (39%) come right after, ahead of other opportunistic mycosis pathogens. Notably, four of these pathogens appear as “critical” or “high” priority in the WHO FPPL, reinforcing that the fungi of greatest concern regionally are the same organisms recognized internationally as requiring strategic public health investment.

There was broad availability of classical methods such as microscopy and culture. However, regarding more sophisticated tools like antigen detection (such as GM and β-D-glucan), as well as molecular methods, there are huge gaps, especially in countries with lowest nominal GDP. Using as a reference the previous survey, we observed limited progress, especially in MALDI–TOF–MS, serologies, and antigen detection^2^. The correlation between higher GDP and the availability of tests such as silver staining and fluorescence microscopy was evident and strongly suggests that there are significant economic barriers preventing access to accurate diagnosis in less privileged countries in the region. Similar associations were also identified with other diagnostic tests. In particular, β-D-glucan had availability below 16%, limiting the possibility of early diagnosis in high-risk patients, especially those with hematological conditions. This contrasts sharply with a recent survey from the Asia/Pacific region, where the availability of β-D-glucan is broader (40%)^12^. The WHO FPPL also highlights inadequate diagnostic access as a core driver of poor outcomes, and our findings demonstrate that these barriers persist in LAC despite global recognition of their urgency.

Our findings reveal a persistent link between national economic status and access to fungal diagnostics and antifungal therapies. Despite the high burden of histoplasmosis among PLWHA, the *Histoplasma* spp. antigen test remains scarce. While many centers have β-D-glucan and GM assays, these remain underutilized, suggesting that availability alone does not ensure effective use. Barriers may include logistical challenges, limited training, or intermittent reagent shortages. Centers in countries with GDP > $10,000 report greater use of advanced diagnostics—GM, *Histoplasma* antigen, and molecular assays—and broader antifungal access. In contrast, under-resourced settings face delays or missed diagnoses, particularly for endemic mycoses like histoplasmosis; systematic antigen testing could improve early detection and reduce mortality^15,16^. Similar GDP-related disparities have been observed in Asia/Pacific, despite existing recommendations^10,12,17–24^. Molecular diagnostics are still mostly outsourced, causing delays that can affect outcomes. Strengthening in-house diagnostic capacity, especially for regionally important pathogens, should be a priority. This gap is consistent with WHO FPPL priorities, which explicitly call for the expansion of diagnostic capacity for high-burden endemic fungi in low- and middle-income regions.

While fluconazole is nearly universally available, access to other essential antifungals is limited: only 21.5% of centers have flucytosine and 29.2% posaconazole. Liposomal amphotericin B—essential for cryptococcosis and histoplasmosis^18,23^—is available in just 60% of centers, often restricted by high costs. Breaking these barriers is crucial, especially in LAC and Africa, where histoplasmosis and cryptococcosis have the greatest impact^2,25,26^. Government initiatives like the Brazilian Endemic Mycoses Program are addressing these gaps, providing flucytosine, liposomal amphotericin B, voriconazole, isavuconazole, and echinocandins. Yet, echinocandins remain poorly accessible in other LAC countries due to formulary and regulatory restrictions. Similar disparities exist in Asia/Pacific, where access to newer antifungals and lipid amphotericin B also depends on national wealth^12^. The WHO FPPL emphasizes the need to include essential antifungals such as flucytosine and lipid amphotericin formulations in national procurement and reimbursement lists, highlighting a concrete area for policy alignment in LAC.

Although centers caring for PLWHA were better equipped than those serving non-HIV populations, significant gaps remain. Notably, 22.8% lacked *Cryptococcus* antigen testing, 45.2% did not offer *Histoplasma* spp. antigen tests, 65.0% had no PCR-based diagnostics for *Pneumocystis jirovecii*, and 76.7% lacked flucytosine, essential for cryptococcal meningitis. These findings highlight that even in relatively well-resourced settings, critical tools for effective fungal infection management are still insufficient or underutilized.

TDM, crucial for optimizing triazole therapy and reducing toxicity, is available in only one-third of surveyed centers. Most lack in-house TDM, leading to delays and suboptimal outcomes, particularly in pediatric and hematologic patients. Similar limitations have been reported in Asia/Pacific and Africa, highlighting a global need for investment in laboratory infrastructure and workforce training^12,27^.

Centers in lower-GDP countries reported a lower IFD burden, likely reflecting underdiagnosis rather than true incidence. This highlights the urgent need for improved surveillance, greater awareness, and integration of fungal disease reporting into national health systems, especially in HIV-endemic regions where cryptococcal meningitis and histoplasmosis remain under-recognized.

Our results are in line with recent global surveys from Africa^26^, Asia/Pacific^12^, and Europe^13^, all of which emphasize the central role of economic resources in shaping the capacity for IFD diagnosis and treatment. The WHO’s Fungal Pathogen Priority List and Essential Medicines List^9,10^, along with ECMM global guidelines^18–23^, offer a framework for minimum standards that are still not universally met in LAC region. To address these gaps, we support establishing reference laboratories and networks for advanced diagnostics and training; public procurement to improve access to essential antifungals; integration of IFD care into existing TB and HIV programs; multicenter studies to better quantify IFD burden; and advocacy for including fungal diagnostics and antifungals in national essential lists.

Our study has several limitations, including potential response bias toward better-resourced centers, variable response rates across countries, and uneven geographical representation, particularly from Brazil, Colombia, Honduras, and Peru. Methodological differences with the 2019 survey preclude direct comparisons. Reliance on self-reported data may introduce reporting bias, and the survey did not capture detailed clinical outcomes or patient-level information, limiting links between access and impact. Participation from Caribbean island countries was limited, primarily affecting smaller island states with relatively low populations. In contrast, larger Caribbean and continental territories, including Mexico, Central America, Colombia, Venezuela, and Guyana, were well represented. Therefore, while the survey provides a robust overview of Latin America and much of the Caribbean, findings may not fully capture diagnostic and therapeutic capacities in the smaller island nations with distinct structural constraints. Finally, while differences by type of institution could provide valuable insights, such an analysis was not feasible due to the heterogeneity of healthcare systems across countries, limited detail on institutional characteristics in our dataset, and the need for dedicated, context-specific studies to accurately identify local bottlenecks. Despite these limitations, the large sample size and broad regional coverage support policy development and advocacy. Notably, molecular diagnostics (42.6%) and β-D-glucan testing (15.9%) were reportedly widely available, though actual routine access may be lower, highlighting the gap between theoretical capacity and real-world implementation.

Significant challenges persist in the diagnosis and management of IFD in LAC. Addressing them will require coordinated regional and international action, investment in laboratory infrastructure, and sustained advocacy to ensure timely access to life-saving diagnostics and therapies. Collaboration among key stakeholders—including ECMM, ESCMID–EFISG, GAFFI, ISHAM, PAHO, WHO, and relevant societies—will be essential to expand diagnostic capacity, improve antifungal access, and integrate fungal diseases into national health agendas. Establishing regional training hubs and promoting cross-country collaboration will support sustainable, equitable solutions. Improving fungal disease management in LAC goes beyond acquiring new technologies; it requires timely implementation, ongoing professional education, and persistent advocacy to reduce disparities across the region. Importantly, our findings align closely with priority actions outlined in the WHO FPPL, which emphasizes the need to strengthen surveillance systems, secure reliable supply chains for essential antifungals (including flucytosine and liposomal amphotericin B), expand access to rapid and accurate diagnostics, and integrate fungal disease management into existing HIV, TB, and transplant care programs. By explicitly linking regional policies and procurement strategies to FPPL recommendations, LAC countries can accelerate progress toward reducing preventable deaths from high-burden fungal infections and ensure greater equity in access to essential care across diverse healthcare settings.

## Method

We conducted a cross-sectional online survey between April 2023 and May 2025 to evaluate the current landscape of fungal diagnostics and access to antifungal therapies across LAC. A structured questionnaire used in earlier international initiatives was employed^2,6,7,12,13,26^. The questionnaire was distributed via a secure web-based platform (EFS, TIVIAN GmbH, Cologne, Germany), managed by the University of Cologne, Cologne, Germany. It was made available in English, French, Portuguese, and Spanish to ensure broad regional accessibility. These four languages are either the primary, widely spoken, or official languages across all countries and territories in LAC, with the exception of Dutch-speaking countries and territories such as Aruba, Bonaire, Curaçao, Saba, Sint Eustatius, Sint Maarten, and Suriname.

Eligible institutions included hospitals, clinical laboratories, and reference centers involved in IFD diagnosis or management. National focal points, identified through professional networks, scientific societies, and organizations. Each site received up to five reminders to maximize participation. Participation was voluntary, required electronic informed consent, and data were anonymized. The research was conducted in close collaboration with local researchers throughout all stages of the process, including study design, implementation, data stewardship, and authorship, ensuring local relevance as jointly determined with local partners. Roles and responsibilities were agreed in advance among collaborators, and discussions included opportunities for capacity building for local researchers. The study would not have been restricted or prohibited in the researchers’ settings. Ethical approval was obtained from the Institutional Review Board (IRB) of the Brazilian institution for the Brazilian authors, as required; approval from other participating countries was not applicable because the study did not involve human or animal subjects, and all data used were derived from publicly available information, such as annual reports provided by participating institutions. Considerations related to animal welfare, environmental protection, biorisk, researcher safety, and benefit sharing were not applicable. The research does not pose risks of stigmatization, discrimination, or personal harm to participants. Finally, relevant local and regional research was appropriately considered and cited throughout the study.

Responses were reviewed for coherence and completeness; incomplete data were included only in relevant analyses. The questionnaire covered pathogen relevance, basic and advanced mycological diagnostics, access to imaging and invasive procedures, antifungal drug availability, and therapeutic drug monitoring, benchmarked against ECMM minimum standards^28^.

Data were exported to SPSS for statistical analysis (IBM Corp., Armonk, New York, United States). Descriptive statistics summarized institutional characteristics and practices. Comparative analyses considered institutional features (HSCT/SOT programs, PLWHA care), and countries were grouped by 2023 GDP per capita (<US$5000, US$5000–10,000, >US$10,000)^29^. Categorical variables were analyzed using Chi-square or Fisher’s exact tests; continuous variables with Student’s t-test or Mann–Whitney U test. Two-tailed *p* values ≤ 0.05 were considered statistically significant.

### Inclusion and ethics statement

This research was conducted as a multinational collaboration involving investigators and institutional representatives from participating countries across Latin America and the Caribbean. Local researchers were involved throughout the research process, including study design, survey adaptation to regional contexts, dissemination within national networks, interpretation of results, and authorship of the manuscript. Data ownership was shared among collaborating investigators, and intellectual contributions were recognized according to established authorship criteria.

The research question was developed in consultation with regional collaborators and reflects locally identified priorities regarding diagnostic and therapeutic capacity for invasive fungal diseases. The study was designed to generate regionally relevant data to inform public health planning and capacity-building initiatives.

Roles and responsibilities were discussed among collaborators prior to study implementation, including coordination through national focal points. Capacity-building components included engagement of early-career researchers, shared data interpretation workshops, and dissemination planning across participating countries.

The research would not have been restricted or prohibited in the settings of the participating researchers. The study consisted of an institutional-level survey and did not involve patient-level data, biological samples, or experimental interventions.

Ethical approval was obtained from the Institutional Review Board (IRB) of the coordinating institution in Brazil, which reviewed the study protocol and determined that the survey did not involve human subjects research requiring full ethics review because no individual-level data were collected. In participating countries, institutional representatives provided electronic informed consent prior to survey completion. Where required by local regulations, local institutional approval was obtained before participation.

The study did not involve animal research, environmental sampling, or activities subject to biorisk regulations. No biological materials were transferred across borders.

The research does not result in stigmatization, incrimination, or personal risk to participants. Data were collected at institutional level only and were anonymized prior to analysis.

The study did not involve health, safety, or security risks to researchers beyond routine academic collaboration.

No biological materials, cultural artifacts, or traditional knowledge were transferred outside participating countries; therefore, benefit-sharing agreements were not applicable.

Local and regional research relevant to invasive fungal diseases in Latin America and the Caribbean was considered and cited where appropriate in the manuscript.

The Institute of Translational Medicine, CECAD, Faculty of Medicine, University of Cologne, Cologne, Germany, covered all costs related to this study, including software expenses, statistical analysis, and publication fees.

### Consideration of sex and gender

This study evaluated institutional diagnostic and therapeutic capacity and did not collect individual-level patient data. As such, sex and gender were not variables in the study design. No analyses stratified by sex or gender were performed because the unit of analysis was the institution rather than individual patients.

### Reporting summary

Further information on research design is available in the Nature Portfolio Reporting Summary linked to this article.

## Supplementary information

Supplementary information

Reporting Summary

Transparent Peer Review file

## Data Availability

All aggregated data supporting the findings of this study are included in the manuscript and its Supplementary Information. Anonymized institutional-level survey data are available from the corresponding author, subject to applicable data protection regulations and institutional agreements. The study did not use publicly archived datasets, accession-based repositories, or third-party restricted clinical databases.

## Code availability

No custom algorithms or unpublished software were developed for this study. Data were analyzed using standard statistical software (SPSS, IBM Corp., Armonk, NY, USA). No custom code central to the findings was generated.
