# Supplementary material for: Fungal diagnostics and antifungal drug access in Latin America and the Caribbean: an ESCMID EFISG multinational survey
Source: Nat Commun. 2026 Jul 28;17:7552. doi: 10.1038/s41467-026-73165-2 (PMC13415557; doi:10.1038/s41467-026-73165-2)
Supplement: Supplementary file 1 — Supplementary information [file 41467_2026_73165_MOESM1_ESM.pdf]

SUPPLEMENTARY INFORMATION

Supplementary table 1. Perceived incidence. high-risk fungal pathogens. and access to diagnostic tools and antifungal treatments in Latin America and the Caribbean, per country.

	**AR**		**BO**		**BR**		**CL**		**CO**		**CR**		**CU**		**DO**		**EC**		**SV**		**GT**	
	n=30		n=6		n=88		n=11		n=205		n=3		n=10		n=7		n=15		n=2		n=11	
	n	*%*	*n*	*%*	*n*	*%*	*n*	*%*	*n*	*%*	*n*	*%*	*n*	*%*	*n*	*%*	*n*	*%*	*n*	*%*	*n*	*%*
**Fungi perceived as of highest risk**																						
Aspergillus spp.	23/30	76.7	2/6	33.3	55/88	62.5	9/11	81.8	91/205	44.4	1/3	33.3	3/10	30.0	7/7	100.0	9/15	60.0	1/2	50.0	5/11	45.5
Candida spp.	26/30	86.7	5/6	83.3	79/88	89.8	10/11	90.9	195/205	95.1	3/3	100.0	10/10	100.0	7/7	100.0	15/15	100.0	2/2	100.0	6/11	54.5
Cryptococcus spp.	24/30	80.0	4/6	66.7	56/88	63.6	5/11	45.5	102/205	49.8	1/3	33.3	3/10	30.0	2/7	28.6	9/15	60.0	1/2	50.0	8/11	72.7
Fusarium spp.	12/30	40.0	0/6	0.0	13/88	14.8	1/11	9.1	21/205	10.2	1/3	33.3	0/10	0.0	1/7	14.3	3/15	20.0	0/2	0.0	0/11	0.0
Histoplasma spp.	20/30	66.7	2/6	33.3	38/88	43.2	2/11	18.2	79/205	38.5	1/3	33.3	0/10	0.0	0/7	0.0	8/15	53.3	2/2	100.0	9/11	81.8
Lomentospora/Scedosporium spp.	1/24	4.2	0/6	0.0	0/81	0.0	1/9	11.1	2/203	1.0	0/2	0.0	0/10	0.0	1/7	14.3	0/15	0.0	0/2	0.0	0/9	0.0
Mucorales	7/30	23.3	1/6	16.7	12/88	13.6	4/11	36.4	22/205	10.7	1/3	33.3	0/10	0.0	1/7	14.3	1/15	6.7	1/2	50.0	3/11	27.3
Phaeohyphomycetes	2/24	8.3	0/6	0.0	3/81	3.7	1/9	11.1	6/203	3.0	0/2	0.0	0/10	0.0	1/7	14.3	1/15	6.7	0/2	0.0	0/9	0.0
**IFI incidence self-perception**																						
Very low	6/30	20.0	3/6	50.0	11/88	12.5	0/10	0.0	81/205	39.5	1/3	33.3	3/10	30.0	1/7	14.3	3/15	20.0	0/2	0.0	1/11	9.1
Low	5/30	16.7	1/6	16.7	23/88	26.1	3/10	30.0	43/205	21.0	2/3	66.7	2/10	20.0	4/7	57.1	3/15	20.0	0/2	0.0	1/11	9.1
Mild	16/30	53.3	2/6	33.3	31/88	35.2	6/10	60.0	61/205	29.8	0/3	0.0	4/10	40.0	2/7	28.6	8/15	53.3	1/2	50.0	4/11	36.4
High	1/30	3.3	0/6	0.0	17/88	19.3	1/10	10.0	16/205	7.8	0/3	0.0	1/10	10.0	0/7	0.0	0/15	0.0	0/2	0.0	4/11	36.4
Very high	2/30	6.7	0/6	0.0	6/88	6.8	0/10	0.0	4/205	2.0	0/3	0.0	0/10	0.0	0/7	0.0	1/15	6.7	1/2	50.0	1/11	9.1
**Microscopy**	29/29	100.0	4/5	80.0	78/80	97.5	10/10	100.0	113/122	92.6	3/3	100.0	10/10	100.0	6/7	85.7	12/13	92.3	2/2	100.0	10/11	90.9
Stains																						
Calcofluor white	6/26	23.1	1/3	33.3	9/45	20.0	4/8	50.0	8/20	40.0	2/3	66.7	2/10	20.0	2/5	40.0	0/10	0.0	1/2	50.0	5/10	50.0
Giemsa	28/28	100.0	2/4	50.0	36/58	62.1	7/9	77.8	38/121	31.4	3/3	100.0	2/10	20.0	5/6	83.3	10/13	76.9	2/2	100.0	8/11	72.7
China/India ink	29/29	100.0	4/5	80.0	78/80	97.5	8/10	80.0	90/122	73.8	3/3	100.0	8/10	80.0	6/7	85.7	12/13	92.3	2/2	100.0	10/11	90.9
KOH	23/27	85.2	1/3	33.3	53/64	82.8	8/8	100.0	108/121	89.3	3/3	100.0	10/10	100.0	5/7	71.4	10/13	76.9	1/2	50.0	6/11	54.5
Silver	13/29	44.8	1/4	25.0	40/65	61.5	5/8	62.5	21/121	17.4	1/3	33.3	0/10	0.0	3/6	50.0	3/13	23.1	1/2	50.0	4/11	36.4
Acces to flouresence	6/29	20.7	0/5	0.0	14/80	17.5	4/10	40.0	11/122	9.0	1/3	33.3	0/10	0.0	3/7	42.9	2/13	15.4	1/2	50.0	2/11	18.2
If suspicion of…																						
Cryptococcosis: Direct exam of body fluids	29/29	100.0	4/5	80.0	77/80	96.3	8/10	80.0	90/90	100.0	3/3	100.0	8/10	80.0	6/7	85.7	11/12	91.7	2/2	100.0	10/11	90.9
Pneumocystosis: Silver staining	13/29	44.8	1/5	20.0	36/80	45.0	3/10	30.0	26/122	21.3	1/3	33.3	0/10	0.0	1/7	14.3	3/13	23.1	1/2	50.0	2/11	18.2
Mucormycosis: Direct microscopy	4/25	16.0	0/5	0.0	20/76	26.3	5/9	55.6	8/122	6.6	1/2	50.0	0/10	0.0	1/7	14.3	0/13	0.0	1/2	50.0	1/10	10.0
**Culture**	28/28	100.0	4/5	80.0	77/77	100.0	10/10	100.0	96/119	80.7	3/3	100.0	10/10	100.0	7/7	100.0	10/13	76.9	2/2	100.0	9/10	90.0
Culture media																						
Agar Niger	5/19	26.3	1/4	25.0	26/47	55.3	5/8	62.5	3/19	15.8	0/3	0.0	1/10	10.0	1/3	33.3	1/8	12.5	1/2	50.0	1/7	14.3
Chromogen	22/22	100.0	2/4	50.0	30/44	68.2	5/6	83.3	56/114	49.1	2/2	100.0	2/10	20.0	2/4	50.0	7/10	70.0	1/2	50.0	2/6	33.3

	AR n=30		BO n=6		BR n=88		CL n=11		CO n=205		CR n=3		CU n=10		DO n=7		EC n=15		SV n=2		GT n=11	
	n	*%*	*n*	*%*	*n*	*%*	*n*	*%*	*n*	*%*	*n*	*%*	*n*	*%*	*n*	*%*	*n*	*%*	*n*	*%*	*n*	*%*
Lactrimel	20/25	80.0	1/3	33.3	11/41	26.8	4/6	66.7	3/110	2.7	2/3	66.7	2/9	22.2	0/3	0.0	3/11	27.3	0/2	0.0	0/7	0.0
Potato agar	20/25	80.0	1/3	33.3	29/44	65.9	7/9	77.8	26/113	23.0	3/3	100.0	2/10	20.0	1/3	33.3	4/10	40.0	2/2	100.0	4/8	50.0
Saboraud agar	25/26	96.2	2/4	50.0	53/59	89.8	7/8	87.5	64/117	54.7	3/3	100.0	8/10	80.0	7/7	100.0	7/10	70.0	2/2	100.0	6/8	75.0
Saboraud agar + Chloramphenicol	21/25	84.0	1/3	33.3	35/47	74.5	5/6	83.3	44/112	39.3	2/3	66.7	10/10	100.0	2/4	50.0	5/10	50.0	2/2	100.0	1/8	12.5
Saboraud agar + Gentamicine	16/25	64.0	0/3	0.0	26/47	55.3	2/6	33.3	24/112	21.4	2/3	66.7	7/10	70.0	1/4	25.0	2/10	20.0	0/2	0.0	1/8	12.5
Selective agar (Chloramphenicol + Cycloheximide)	16/25	64.0	1/3	33.3	26/45	57.8	3/6	50.0	17/113	15.0	3/3	100.0	4/10	40.0	2/4	50.0	2/10	20.0	2/2	100.0	3/7	42.9
Available tests for species identification	28/28	100.0	3/4	75.0	70/72	97.2	10/10	100.0	72/117	61.5	3/3	100.0	6/9	66.7	6/6	100.0	10/12	83.3	1/2	50.0	8/9	88.9
Automated identification	20/28	71.4	0/4	0.0	57/71	80.3	6/8	75.0	37/117	31.6	2/3	66.7	2/9	22.2	6/6	100.0	7/12	58.3	1/2	50.0	6/7	85.7
Biochemical tests	26/28	92.9	3/4	75.0	45/64	70.3	8/9	88.9	23/115	20.0	1/3	33.3	6/9	66.7	4/6	66.7	6/11	54.5	1/2	50.0	6/9	66.7
DNA sequencing	8/27	29.6	0/4	0.0	16/66	24.2	6/9	66.7	23/117	19.7	1/3	33.3	1/9	11.1	0/3	0.0	2/11	18.2	0/2	0.0	2/6	33.3
MALDI–TOF–MS	15/28	53.6	0/4	0.0	31/68	45.6	7/9	77.8	16/117	13.7	2/3	66.7	1/9	11.1	3/6	50.0	2/11	18.2	0/2	0.0	2/7	28.6
Mounting medium	7/22	31.8	0/4	0.0	16/51	31.4	3/6	50.0	36/111	32.4	1/3	33.3	0/9	0.0	0/5	0.0	3/10	30.0	1/2	50.0	0/7	0.0
Available antifungal susceptibility test technologies	27/28	96.4	3/5	60.0	63/77	81.8	9/10	90.0	80/119	67.2	3/3	100.0	6/10	60.0	6/6	100.0	10/13	76.9	1/2	50.0	8/10	80.0
Broth microdilution. using CLSI standards	17/24	70.8	2/4	50.0	23/53	43.4	5/7	71.4	16/115	13.9	2/3	66.7	1/10	10.0	2/4	50.0	5/12	41.7	0/2	0.0	0/6	0.0
Broth microdilution. using EUCAST standards	9/24	37.5	1/4	25.0	22/57	38.6	4/7	57.1	4/112	3.6	0/3	0.0	0/10	0.0	1/3	33.3	2/11	18.2	0/2	0.0	0/6	0.0
Gradient diffusion test	16/24	66.7	1/4	25.0	19/50	38.0	5/8	62.5	14/114	12.3	3/3	100.0	3/10	30.0	4/6	66.7	2/9	22.2	0/2	0.0	0/6	0.0
Automated identification	13/27	48.1	0/4	0.0	40/62	64.5	5/8	62.5	63/117	53.8	2/3	66.7	1/10	10.0	6/6	100.0	7/11	63.6	1/2	50.0	5/7	71.4
**Serology**	26/26	100.0	2/4	50.0	62/72	86.1	7/10	70.0	23/27	85.2	2/3	66.7	2/10	20.0	5/5	100.0	6/10	60.0	1/2	50.0	7/9	77.8
Aspergillus spp.	26/26	100.0	1/4	25.0	57/72	79.2	7/10	70.0	21/26	80.8	2/3	66.7	1/10	10.0	5/5	100.0	6/10	60.0	1/2	50.0	7/9	77.8
Onsite	19/26	73.1	0/4	0.0	28/72	38.9	4/10	40.0	11/26	42.3	0/3	0.0	0/10	0.0	3/5	60.0	2/10	20.0	0/2	0.0	4/9	44.4
Outsourced	7/26	26.9	1/4	25.0	29/72	40.3	3/10	30.0	10/26	38.5	2/3	66.7	1/10	10.0	2/5	40.0	4/10	40.0	1/2	50.0	3/9	33.3
Candida spp.	11/26	42.3	2/4	50.0	18/65	27.7	5/10	50.0	9/23	39.1	2/3	66.7	1/10	10.0	3/5	60.0	6/10	60.0	1/2	50.0	3/9	33.3
Onsite	6/26	23.1	2/4	50.0	8/65	12.3	2/10	20.0	3/23	13.0	0/3	0.0	0/10	0.0	3/5	60.0	2/10	20.0	0/2	0.0	1/9	11.1
Outsourced	5/26	19.2	0/4	0.0	10/65	15.4	3/10	30.0	6/23	26.1	2/3	66.7	1/10	10.0	0/5	0.0	4/10	40.0	1/2	50.0	2/9	22.2
Histoplasma spp.	25/26	96.2	2/4	50.0	53/71	74.6	5/10	50.0	22/27	81.5	2/3	66.7	2/10	20.0	3/5	60.0	5/9	55.6	1/2	50.0	7/9	77.8
Onsite	18/26	69.2	1/4	25.0	27/71	38.0	2/10	20.0	10/27	37.0	0/3	0.0	0/10	0.0	2/5	40.0	2/9	22.2	0/2	0.0	5/9	55.6
Outsourced	7/26	26.9	1/4	25.0	26/71	36.6	3/10	30.0	12/27	44.4	2/3	66.7	2/10	20.0	1/5	20.0	3/9	33.3	1/2	50.0	2/9	22.2
Paracoccidioides spp.	24/26	92.3	1/4	25.0	53/69	76.8	1/9	11.1	14/24	58.3	2/3	66.7	0/10	0.0	1/5	20.0	5/9	55.6	1/2	50.0	3/9	33.3
Onsite	17/26	65.4	0/4	0.0	26/69	37.7	0/9	0.0	3/24	12.5	0/3	0.0	0/10	0.0	0/5	0.0	2/9	22.2	0/2	0.0	1/9	11.1
Outsourced	7/26	26.9	1/4	25.0	27/69	39.1	1/9	11.1	11/24	45.8	2/3	66.7	0/10	0.0	1/5	20.0	3/9	33.3	1/2	50.0	2/9	22.2
**Antigen detection**	25/25	100.0	3/4	75.0	66/70	94.3	10/10	100.0	77/116	66.4	3/3	100.0	4/10	40.0	6/6	100.0	6/12	50.0	1/2	50.0	9/9	100.0
Aspergillus spp. GM	23/25	92.0	1/4	25.0	54/67	80.6	10/10	100.0	69/115	60.0	2/3	66.7	2/10	20.0	6/6	100.0	5/12	41.7	1/2	50.0	8/9	88.9
Aspergillus spp. GM (ELISA)	18/25	72.0	1/4	25.0	37/60	61.7	9/10	90.0	55/62	88.7	1/2	50.0	2/10	20.0	4/6	66.7	5/11	45.5	1/2	50.0	4/8	50.0

	AR n=30		BO n=6		BR n=88		CL n=11		CO n=205		CR n=3		CU n=10		DO n=7		EC n=15		SV n=2		GT n=11	
	n	*%*	*n*	*%*	*n*	*%*	*n*	*%*	*n*	*%*	*n*	*%*	*n*	*%*	*n*	*%*	*n*	*%*	*n*	*%*	*n*	*%*
Onsite	16/25	64.0	0/4	0.0	14/60	23.3	6/10	60.0	20/62	32.3	0/2	0.0	0/10	0.0	3/6	50.0	1/11	9.1	0/2	0.0	1/8	12.5
Outsourced	2/25	8.0	1/4	25.0	23/60	38.3	3/10	30.0	35/62	56.5	1/2	50.0	2/10	20.0	1/6	16.7	4/11	36.4	1/2	50.0	3/8	37.5
Aspergillus spp. GM (LFA)	16/25	64.0	1/4	25.0	24/55	43.6	5/10	50.0	49/114	43.0	1/3	33.3	2/10	20.0	2/6	33.3	3/11	27.3	1/2	50.0	5/9	55.6
Onsite	12/25	48.0	0/4	0.0	10/55	18.2	2/10	20.0	14/114	12.3	0/3	0.0	0/10	0.0	0/6	0.0	1/11	9.1	0/2	0.0	2/9	22.2
Outsourced	4/25	16.0	1/4	25.0	14/55	25.5	3/10	30.0	35/114	30.7	1/3	33.3	2/10	20.0	2/6	33.3	2/11	18.2	1/2	50.0	3/9	33.3
Aspergillus spp. GM (LFD)	16/25	64.0	1/4	25.0	33/62	53.2	4/10	40.0	13/25	52.0	1/3	33.3	2/10	20.0	4/6	66.7	3/10	30.0	1/2	50.0	6/8	75.0
Onsite	12/25	48.0	0/4	0.0	17/62	27.4	2/10	20.0	7/25	28.0	0/3	0.0	0/10	0.0	1/6	16.7	0/10	0.0	0/2	0.0	5/8	62.5
Outsourced	4/25	16.0	1/4	25.0	16/62	25.8	2/10	20.0	6/25	24.0	1/3	33.3	2/10	20.0	3/6	50.0	3/10	30.0	1/2	50.0	1/8	12.5
Candida spp.	1/25	4.0	1/4	25.0	11/60	18.3	2/10	20.0	35/115	30.4	2/3	66.7	1/10	10.0	2/5	40.0	3/12	25.0	1/2	50.0	3/8	37.5
Onsite	0/25	0.0	1/4	25.0	2/60	3.3	0/10	0.0	6/115	5.2	0/3	0.0	0/10	0.0	1/5	20.0	0/12	0.0	0/2	0.0	1/8	12.5
Outsourced	1/25	4.0	0/4	0.0	9/60	15.0	2/10	20.0	29/115	25.2	2/3	66.7	1/10	10.0	1/5	20.0	3/12	25.0	1/2	50.0	2/8	25.0
Cryptococcus spp. GM	23/25	92.0	2/4	50.0	60/69	87.0	10/10	100.0	75/116	64.7	3/3	100.0	4/10	40.0	3/6	50.0	5/11	45.5	1/2	50.0	8/9	88.9
Cryptococcus spp. GM (LAT)	21/25	84.0	2/4	50.0	49/67	73.1	8/10	80.0	56/116	48.3	2/3	66.7	4/10	40.0	2/6	33.3	5/11	45.5	1/2	50.0	5/9	55.6
Onsite	19/25	76.0	1/4	25.0	35/67	52.2	5/10	50.0	20/116	17.2	0/3	0.0	1/10	10.0	0/6	0.0	2/11	18.2	1/2	50.0	3/9	33.3
Outsourced	2/25	8.0	1/4	25.0	14/67	20.9	3/10	30.0	36/116	31.0	2/3	66.7	3/10	30.0	2/6	33.3	3/11	27.3	0/2	0.0	2/9	22.2
Cryptococcus spp. GM (LFA)	19/25	76.0	1/4	25.0	36/64	56.3	6/10	60.0	59/114	51.8	2/3	66.7	2/10	20.0	3/6	50.0	4/11	36.4	1/2	50.0	8/9	88.9
Onsite	18/25	72.0	0/4	0.0	28/64	43.8	5/10	50.0	31/114	27.2	0/3	0.0	1/10	10.0	1/6	16.7	2/11	18.2	1/2	50.0	6/9	66.7
Outsourced	1/25	4.0	1/4	25.0	8/64	12.5	1/10	10.0	28/114	24.6	2/3	66.7	1/10	10.0	2/6	33.3	2/11	18.2	0/2	0.0	2/9	22.2
Histoplasma spp.	18/25	72.0	2/4	50.0	34/67	50.7	7/10	70.0	62/116	53.4	2/3	66.7	2/10	20.0	3/6	50.0	3/11	27.3	1/2	50.0	6/9	66.7
Onsite	15/25	60.0	1/4	25.0	14/67	20.9	2/10	20.0	13/116	11.2	0/3	0.0	0/10	0.0	1/6	16.7	0/11	0.0	1/2	50.0	4/9	44.4
Outsourced	3/25	12.0	1/4	25.0	20/67	29.9	5/10	50.0	49/116	42.2	2/3	66.7	2/10	20.0	2/6	33.3	3/11	27.3	0/2	0.0	2/9	22.2
β-D-glucan	1/25	4.0	2/4	50.0	13/62	21.0	6/10	60.0	0/116	0.0	2/3	66.7	1/10	10.0	3/6	50.0	3/11	27.3	1/2	50.0	1/6	16.7
Onsite	0/25	0.0	1/4	25.0	1/62	1.6	2/10	20.0	0/116	0.0	0/3	0.0	0/10	0.0	1/6	16.7	1/11	9.1	0/2	0.0	0/6	0.0
Outsourced	1/25	4.0	1/4	25.0	12/62	19.4	4/10	40.0	0/116	0.0	2/3	66.7	1/10	10.0	2/6	33.3	2/11	18.2	1/2	50.0	1/6	16.7
**Molecular tests**	16/25	64.0	3/4	75.0	29/66	43.9	9/10	90.0	51/62	82.3	1/2	50.0	1/10	10.0	3/5	60.0	3/10	30.0	1/2	50.0	8/10	80.0
Aspergillus spp.	11/24	45.8	2/4	50.0	19/62	30.6	7/10	70.0	38/54	70.4	1/2	50.0	1/10	10.0	2/5	40.0	3/10	30.0	1/2	50.0	4/10	40.0
Onsite	4/24	16.7	1/4	25.0	8/62	12.9	4/10	40.0	5/54	9.3	1/2	50.0	0/10	0.0	0/5	0.0	1/10	10.0	0/2	0.0	1/10	10.0
Outsourced	7/24	29.2	1/4	25.0	11/62	17.7	3/10	30.0	33/54	61.1	0/2	0.0	1/10	10.0	2/5	40.0	2/10	20.0	1/2	50.0	3/10	30.0
Candida spp.	8/25	32.0	2/4	50.0	14/60	23.3	4/9	44.4	39/54	72.2	0/2	0.0	1/10	10.0	2/5	40.0	3/10	30.0	1/2	50.0	5/10	50.0
Onsite	3/25	12.0	1/4	25.0	5/60	8.3	2/9	22.2	12/54	22.2	0/2	0.0	0/10	0.0	1/5	20.0	1/10	10.0	0/2	0.0	2/10	20.0
Outsourced	5/25	20.0	1/4	25.0	9/60	15.0	2/9	22.2	27/54	50.0	0/2	0.0	1/10	10.0	1/5	20.0	2/10	20.0	1/2	50.0	3/10	30.0
Pneumocystis jirovecii	14/25	56.0	3/4	75.0	24/66	36.4	9/10	90.0	45/58	77.6	0/2	0.0	1/10	10.0	3/5	60.0	3/10	30.0	1/2	50.0	7/10	70.0
Onsite	8/25	32.0	2/4	50.0	12/66	18.2	5/10	50.0	8/58	13.8	0/2	0.0	0/10	0.0	0/5	0.0	1/10	10.0	0/2	0.0	3/10	30.0
Outsourced	6/25	24.0	1/4	25.0	12/66	18.2	4/10	40.0	37/58	63.8	0/2	0.0	1/10	10.0	3/5	60.0	2/10	20.0	1/2	50.0	4/10	40.0
Mucorales	5/20	25.0	1/4	25.0	12/56	21.4	3/8	37.5	34/50	68.0	0/1	0.0	1/10	10.0	2/5	40.0	3/10	30.0	1/2	50.0	3/8	37.5
Onsite	1/20	5.0	0/4	0.0	3/56	5.4	1/8	12.5	3/50	6.0	0/1	0.0	0/10	0.0	0/5	0.0	1/10	10.0	0/2	0.0	0/8	0.0
Outsourced	4/20	20.0	1/4	25.0	9/56	16.1	2/8	25.0	31/50	62.0	0/1	0.0	1/10	10.0	2/5	40.0	2/10	20.0	1/2	50.0	3/8	37.5
**Imaging procedures**																						
CT	13/17	76.5	1/4	25.0	62/65	95.4	8/8	100.0	80/100	80.0	0/2	0.0	2/10	20.0	6/6	100.0	6/11	54.5	0/2	0.0	4/8	50.0
PET CT	2/17	11.8	0/4	0.0	18/65	27.7	3/8	37.5	24/100	24.0	1/2	50.0	0/10	0.0	2/6	33.3	2/11	18.2	0/2	0.0	0/8	0.0

	AR		BO		BR		CL		CO		CR		CU		DO		EC		SV		GT	
	n=30		n=6		n=88		n=11		n=205		n=3		n=10		n=7		n=15		n=2		n=11	
	n	*%*	*n*	*%*	*n*	*%*	*n*	*%*	*n*	*%*	*n*	*%*	*n*	*%*	*n*	*%*	*n*	*%*	*n*	*%*	*n*	*%*
MRI	9/17	52.9	1/4	25.0	51/65	78.5	6/8	75.0	62/100	62.0	0/2	0.0	0/10	0.0	6/6	100.0	5/11	45.5	0/2	0.0	0/8	0.0
PET MRI	2/17	11.8	0/4	0.0	2/65	3.1	0/8	0.0	9/100	9.0	1/2	50.0	0/10	0.0	0/6	0.0	2/11	18.2	0/2	0.0	0/8	0.0
Ultrasound	14/17	82.4	3/4	75.0	60/65	92.3	8/8	100.0	87/100	87.0	1/2	50.0	9/10	90.0	6/6	100.0	10/11	90.9	0/2	0.0	5/8	62.5
X ray	12/17	70.6	3/4	75.0	59/65	90.8	7/8	87.5	84/100	84.0	1/2	50.0	9/10	90.0	5/6	83.3	7/11	63.6	1/2	50.0	6/8	75.0
**Surgery**	11/16	68.8	1/1	100.0	50/57	87.7	6/6	100.0	69/86	80.2	1/2	50.0	6/10	60.0	5/6	83.3	4/10	40.0	1/2	50.0	3/6	50.0
**Systemic antifungals**																						
Amphotericin B	29/29	100.0	4/5	80.0	83/85	97.6	9/9	100.0	87/102	85.3	2/3	66.7	10/10	100.0	7/7	100.0	5/10	50.0	2/2	100.0	10/11	90.9
Deoxycholate	23/26	88.5	3/5	60.0	75/84	89.3	7/8	87.5	65/101	64.4	2/3	66.7	5/10	50.0	5/7	71.4	3/9	33.3	2/2	100.0	9/11	81.8
Lipid-based formulations	27/29	93.1	2/5	40.0	68/84	81.0	9/9	100.0	81/102	79.4	0/3	0.0	6/10	60.0	1/7	14.3	2/9	22.2	1/1	100.0	2/10	20.0
Lipidic complex	10/23	43.5	2/5	40.0	45/80	56.3	0/6	0.0	39/99	39.4	0/3	0.0	0/10	0.0	3/7	42.9	1/8	12.5	0/2	0.0	3/10	30.0
Liposomal	25/28	89.3	1/5	20.0	64/83	77.1	9/9	100.0	77/102	75.5	0/3	0.0	6/10	60.0	5/7	71.4	1/8	12.5	2/2	100.0	6/9	66.7
Echinocandins	22/30	73.3	1/5	20.0	71/84	84.5	9/9	100.0	85/102	83.3	1/3	33.3	0/10	0.0	7/7	100.0	6/11	54.5	0/2	0.0	3/9	33.3
Anidulafungin	16/27	59.3	1/4	25.0	45/81	55.6	7/7	100.0	55/101	54.5	1/3	33.3	0/10	0.0	6/6	100.0	1/10	10.0	0/2	0.0	1/9	11.1
Caspofungin	15/28	53.6	0/4	0.0	21/79	26.6	6/8	75.0	78/101	77.2	1/3	33.3	0/10	0.0	5/7	71.4	6/11	54.5	0/2	0.0	2/9	22.2
Micafungin	4/27	14.8	0/5	0.0	53/82	64.6	4/8	50.0	19/100	19.0	0/3	0.0	0/10	0.0	0/7	0.0	1/10	10.0	0/2	0.0	1/9	11.1
Triazoles	30/30	100.0	6/6	100.0	84/85	98.8	9/9	100.0	99/102	97.1	2/3	66.7	9/10	90.0	7/7	100.0	11/11	100.0	2/2	100.0	11/11	100.0
Fluconazole	30/30	100.0	6/6	100.0	84/85	98.8	9/9	100.0	98/102	96.1	2/3	66.7	9/10	90.0	7/7	100.0	11/11	100.0	2/2	100.0	11/11	100.0
Mould-active triazoles	30/30	100.0	4/5	80.0	80/84	95.2	9/9	100.0	86/102	84.3	2/3	66.7	0/10	0.0	7/7	100.0	9/11	81.8	2/2	100.0	9/10	90.0
Itraconazole	22/30	73.3	4/5	80.0	71/82	86.6	6/8	75.0	52/102	51.0	1/3	33.3	0/10	0.0	6/7	85.7	9/11	81.8	1/2	50.0	7/10	70.0
Isavuconazole	16/28	57.1	2/5	40.0	26/79	32.9	8/8	100.0	60/97	61.9	1/3	33.3	0/10	0.0	0/6	0.0	1/10	10.0	0/2	0.0	3/10	30.0
Posaconazole	12/26	46.2	0/5	0.0	16/78	20.5	5/8	62.5	53/100	53.0	0/3	0.0	0/10	0.0	0/5	0.0	1/11	9.1	1/2	50.0	0/9	0.0
Voriconazole	27/30	90.0	2/5	40.0	60/81	74.1	9/9	100.0	71/101	70.3	1/3	33.3	0/10	0.0	7/7	100.0	7/11	63.6	0/2	0.0	4/10	40.0
Flucytosine	6/28	21.4	0/5	0.0	34/79	43.0	0/7	0.0	42/98	42.9	0/3	0.0	0/10	0.0	0/7	0.0	0/10	0.0	0/2	0.0	2/9	22.2
Terbinafine	13/26	50.0	4/5	80.0	19/77	24.7	4/8	50.0	16/97	16.5	2/3	66.7	1/10	10.0	4/6	66.7	7/11	63.6	0/2	0.0	2/9	22.2
**Therapeutic drug monitoring**	13/23	56.5	1/4	25.0	26/65	40.0	8/10	80.0	35/90	38.9	0/2	0.0	1/10	10.0	2/6	33.3	2/9	22.2	1/2	50.0	3/8	37.5
Flucytosine	0/21	0.0	0/4	0.0	7/59	11.9	0/9	0.0	6/79	7.6	0/2	0.0	0/9	0.0	0/6	0.0	0/7	0.0	0/2	0.0	1/8	12.5
Onsite	0/21	0.0	0/4	0.0	7/59	11.9	0/9	0.0	0/79	0.0	0/2	0.0	0/9	0.0	0/6	0.0	0/7	0.0	0/2	0.0	1/8	12.5
Outsourced	0/21	0.0	0/4	0.0	0/59	0.0	0/9	0.0	6/79	7.6	0/2	0.0	0/9	0.0	0/6	0.0	0/7	0.0	0/2	0.0	0/8	0.0
Isavuconazole	1/15	6.7	1/4	25.0	3/52	5.8	0/7	0.0	10/77	13.0	0/1	0.0	0/10	0.0	0/5	0.0	0/7	0.0	0/2	0.0	0/6	0.0
Onsite	0/15	0.0	1/4	25.0	3/52	5.8	0/7	0.0	3/77	3.9	0/1	0.0	0/10	0.0	0/5	0.0	0/7	0.0	0/2	0.0	0/6	0.0
Outsourced	1/15	6.7	0/4	0.0	0/52	0.0	0/7	0.0	7/77	9.1	0/1	0.0	0/10	0.0	0/5	0.0	0/7	0.0	0/2	0.0	0/6	0.0
Itraconazole	8/22	36.4	1/4	25.0	14/60	23.3	1/8	12.5	23/77	29.9	0/2	0.0	0/8	0.0	1/6	16.7	2/8	25.0	1/2	50.0	3/8	37.5
Onsite	4/22	18.2	1/4	25.0	12/60	20.0	0/8	0.0	5/77	6.5	0/2	0.0	0/8	0.0	1/6	16.7	2/8	25.0	1/2	50.0	3/8	37.5
Outsourced	4/22	18.2	0/4	0.0	2/60	3.3	1/8	12.5	18/77	23.4	0/2	0.0	0/8	0.0	0/6	0.0	0/8	0.0	0/2	0.0	0/8	0.0
Posaconazole	3/19	15.8	0/4	0.0	3/56	5.4	4/7	57.1	17/79	21.5	0/2	0.0	0/10	0.0	0/4	0.0	0/7	0.0	0/2	0.0	0/8	0.0
Onsite	1/19	5.3	0/4	0.0	3/56	5.4	2/7	28.6	4/79	5.1	0/2	0.0	0/10	0.0	0/4	0.0	0/7	0.0	0/2	0.0	0/8	0.0
Outsourced	2/19	10.5	0/4	0.0	0/56	0.0	2/7	28.6	13/79	16.5	0/2	0.0	0/10	0.0	0/4	0.0	0/7	0.0	0/2	0.0	0/8	0.0
Voriconazole	11/23	47.8	0/4	0.0	21/59	35.6	8/9	88.9	28/85	32.9	0/2	0.0	1/10	10.0	2/6	33.3	2/8	25.0	0/2	0.0	1/8	12.5
Onsite	6/23	26.1	0/4	0.0	19/59	32.2	3/9	33.3	6/85	7.1	0/2	0.0	1/10	10.0	2/6	33.3	2/8	25.0	0/2	0.0	1/8	12.5
Outsourced	5/23	21.7	0/4	0.0	2/59	3.4	5/9	55.6	22/85	25.9	0/2	0.0	0/10	0.0	0/6	0.0	0/8	0.0	0/2	0.0	0/8	0.0

AR, Argentina; **BO**, Bolivia; **BR**, Brazil; **CL**, Chile; **CLSI**, Clinical and Laboratory Standards Institute; **CO**, Colombia; **CR**, Costa Rica; **CT**, computed tomography; **CU**, Cuba; **DNA**, deoxyribonucleic acid; **DO**, Dominican Republic; **EC**, Ecuador; **ELISA**, enzyme-linked immunosorbent assay; **EUCAST**, European Committee on Antimicrobial Susceptibility Testing; **GM**, galactomannan; **GT**, Guatemala; **HIV**, human immunodeficiency virus; **HSCT**, hematopoietic stem cell transplantation; **IFD**, invasive fungal disease; **KOH**, potassium hydroxide; **LAT**, latex agglutination test; **LFA**, lateral flow assay; **LFD**, lateral flow device; **MALDI–TOF–MS**, matrix-assisted laser desorption/ionization time-of-flight mass spectrometry; **MRI**, magnetic resonance imaging; **n**, number (sample size); **p**, probability; **PET**, positron emission tomography; **SOT**, solid organ transplantation; **spp.**, species; **SV**, El Salvador

Supplementary table 2. Perceived incidence. high-risk fungal pathogens. and access to diagnostic tools and antifungal treatments in Latin America and the Caribbean, per country.

	**GY**		**HT**		**HN**		**JM**		**MX**		**NI**		**PA**		**PY**		**PE**		**TT**		**UY**		**VE**	
	n=1		n=1		n=80		n=1		n=42		n=1		n=6		n=5		n=80		n=1		n=1		n=12	
	n	*%*	*n*	*%*	*n*	*%*	*n*	*%*	*n*	*%*	*n*	*%*	*n*	*%*	*n*	*%*	*n*	*%*	*n*	*%*	*n*	*%*	*n*	*%*
**Fungi perceived as of highest risk**																								
Aspergillus spp.	1/1	100.0	0/1	0.0	27/80	33.8	1/1	100.0	34/42	81.0	1/1	100.0	2/6	33.3	3/5	60.0	49/80	61.3	1/1	100.0	1/1	100.0	8/12	66.7
Candida spp.	1/1	100.0	1/1	100.0	76/80	95.0	1/1	100.0	38/42	90.5	1/1	100.0	6/6	100.0	5/5	100.0	72/80	90.0	1/1	100.0	1/1	100.0	11/12	91.7
Cryptococcus spp.	1/1	100.0	0/1	0.0	13/80	16.3	1/1	100.0	24/42	57.1	1/1	100.0	3/6	50.0	4/5	80.0	42/80	52.5	1/1	100.0	1/1	100.0	8/12	66.7
Fusarium spp.	0/1	0.0	0/1	0.0	8/80	10.0	0/1	0.0	6/42	14.3	0/1	0.0	0/6	0.0	2/5	40.0	2/80	2.5	1/1	100.0	0/1	0.0	5/12	41.7
Histoplasma spp.	1/1	100.0	0/1	0.0	14/80	17.5	0/1	0.0	24/42	57.1	1/1	100.0	6/6	100.0	4/5	80.0	19/80	23.8	1/1	100.0	1/1	100.0	8/12	66.7
Lomentospora/*Scedosporium* spp.	0/1	0.0	0/1	0.0	3/80	3.8	0/1	0.0	0/37	0.0	0/1	0.0	0/6	0.0	0/4	0.0	0/79	0.0	0/1	0.0	0/1	0.0	2/11	18.2
Mucorales	0/1	0.0	0/1	0.0	10/80	12.5	0/1	0.0	24/42	57.1	1/1	100.0	0/6	0.0	2/5	40.0	13/80	16.3	1/1	100.0	0/1	0.0	6/12	50.0
Phaeohyphomycetes	0/1	0.0	0/1	0.0	4/80	5.0	0/1	0.0	1/37	2.7	0/1	0.0	0/6	0.0	1/4	25.0	1/79	1.3	1/1	100.0	0/1	0.0	2/11	18.2
**IFI incidence self-perception**																								
Very low	0/1	0.0	0/1	0.0	41/79	51.9	0/1	0.0	4/42	9.5	0/1	0.0	1/6	16.7	0/5	0.0	29/80	36.3	0/1	0.0	0/1	0.0	2/12	16.7
Low	1/1	100.0	0/1	0.0	15/79	19.0	1/1	100.0	11/42	26.2	0/1	0.0	3/6	50.0	2/5	40.0	24/80	30.0	1/1	100.0	1/1	100.0	4/12	33.3
Mild	0/1	0.0	1/1	100.0	14/79	17.7	0/1	0.0	21/42	50.0	1/1	100.0	1/6	16.7	3/5	60.0	22/80	27.5	0/1	0.0	0/1	0.0	2/12	16.7
High	0/1	0.0	0/1	0.0	5/79	6.3	0/1	0.0	3/42	7.1	0/1	0.0	1/6	16.7	0/5	0.0	4/80	5.0	0/1	0.0	0/1	0.0	3/12	25.0
Very high	0/1	0.0	0/1	0.0	4/79	5.1	0/1	0.0	3/42	7.1	0/1	0.0	0/6	0.0	0/5	0.0	1/80	1.3	0/1	0.0	0/1	0.0	1/12	8.3
**Microscopy**	1/1	100.0	0/0	0.0	59/65	90.8	1/1	100.0	41/41	100.0	1/1	100.0	5/5	100.0	5/5	100.0	71/72	98.6	1/1	100.0	1/1	100.0	12/12	100.0
Stains																								
Calcofluor white	0/0	0.0	0/0	0.0	0/7	0.0	1/1	100.0	12/37	32.4	0/1	0.0	0/3	0.0	0/3	0.0	3/30	10.0	1/1	100.0	1/1	100.0	1/10	10.0
Giemsa	1/1	100.0	0/0	0.0	16/65	24.6	0/0	0.0	26/39	66.7	1/1	100.0	1/3	33.3	5/5	100.0	50/68	73.5	1/1	100.0	1/1	100.0	12/12	100.0
China/India ink	1/1	100.0	0/0	0.0	27/65	41.5	1/1	100.0	41/41	100.0	1/1	100.0	5/5	100.0	5/5	100.0	64/69	92.8	1/1	100.0	1/1	100.0	11/11	100.0
KOH	1/1	100.0	0/0	0.0	56/62	90.3	1/1	100.0	35/40	87.5	1/1	100.0	2/4	50.0	5/5	100.0	62/67	92.5	1/1	100.0	1/1	100.0	12/12	100.0
Silver	1/1	100.0	0/0	0.0	1/63	1.6	1/1	100.0	19/39	48.7	0/1	0.0	1/3	33.3	1/3	33.3	13/55	23.6	1/1	100.0	1/1	100.0	3/11	27.3
Acces to flouresence	1/1	100.0	0/0	0.0	1/65	1.5	0/1	0.0	8/41	19.5	0/1	0.0	0/5	0.0	1/5	20.0	2/72	2.8	1/1	100.0	1/1	100.0	0/12	0.0
If suspicion of…																								
Cryptococcosis: Direct exam of body fluids	1/1	100.0	0/0	0.0	39/41	95.1	1/1	100.0	41/41	100.0	1/1	100.0	5/5	100.0	5/5	100.0	63/72	87.5	1/1	100.0	1/1	100.0	11/12	91.7
Pneumocystosis: Silver staining	1/1	100.0	0/0	0.0	3/65	4.6	1/1	100.0	13/41	31.7	0/1	0.0	1/5	20.0	1/5	20.0	5/72	6.9	1/1	100.0	1/1	100.0	3/12	25.0
Mucormycosis: Direct microscopy	0/1	0.0	0/0	0.0	6/65	9.2	0/1	0.0	11/40	27.5	0/1	0.0	1/5	20.0	2/4	50.0	2/71	2.8	0/1	0.0	0/1	0.0	5/12	41.7
**Culture**	1/1	100.0	0/0	0.0	46/60	76.7	1/1	100.0	41/42	97.6	1/1	100.0	5/5	100.0	4/4	100.0	60/67	89.6	1/1	100.0	1/1	100.0	11/12	91.7
Culture media																								
Agar Niger	1/1	100.0	0/0	0.0	2/10	20.0	0/0	0.0	8/38	21.1	0/1	0.0	0/4	0.0	2/3	66.7	6/32	18.8	0/0	0.0	0/1	0.0	1/12	8.3
Chromogen	1/1	100.0	0/0	0.0	15/59	25.4	0/1	0.0	30/38	78.9	0/1	0.0	3/5	60.0	2/2	100.0	20/50	40.0	0/0	0.0	1/1	100.0	8/11	72.7
Lactrimel	1/1	100.0	0/0	0.0	2/58	3.4	0/0	0.0	2/38	5.3	0/1	0.0	0/3	0.0	3/3	100.0	3/43	7.0	0/0	0.0	0/1	0.0	8/12	66.7
Potato agar	1/1	100.0	0/0	0.0	2/58	3.4	1/1	100.0	19/40	47.5	0/1	0.0	2/4	50.0	3/3	100.0	11/46	23.9	1/1	100.0	1/1	100.0	8/12	66.7

	GY n=1		HT n=1		HN n=80		JM n=1		MX n=42		NI n=1		PA n=6		PY n=5		PE n=80		TT n=1		UY n=1		VE n=12	
	n	%	*n*	%	*n*	%	*n*	%	*n*	%	*n*	%	*n*	%	*n*	%	*n*	%	*n*	%	*n*	%	*n*	%
Saboraud agar	0/0	0.0	0/0	0.0	35/59	59.3	1/1	100.0	39/41	95.1	1/1	100.0	4/5	80.0	4/4	100.0	52/61	85.2	1/1	100.0	1/1	100.0	10/12	83.3
Saboraud agar + Chloramphenicol	1/1	100.0	0/0	0.0	10/58	17.2	0/0	0.0	12/38	31.6	1/1	100.0	0/4	0.0	3/3	100.0	25/44	56.8	1/1	100.0	1/1	100.0	8/12	66.7
Saboraud agar + Gentamicine	0/0	0.0	0/0	0.0	6/58	10.3	0/0	0.0	7/38	18.4	0/1	0.0	0/4	0.0	3/3	100.0	9/41	22.0	1/1	100.0	0/1	0.0	7/12	58.3
Selective agar (Chloramphenicol + Cycloheximide)	0/0	0.0	0/0	0.0	5/58	8.6	0/0	0.0	13/39	33.3	0/1	0.0	1/4	25.0	3/3	100.0	9/39	23.1	0/0	0.0	1/1	100.0	9/12	75.0
Available tests for species identification	1/1	100.0	0/0	0.0	20/60	33.3	1/1	100.0	41/41	100.0	1/1	100.0	5/5	100.0	4/4	100.0	52/64	81.3	1/1	100.0	1/1	100.0	9/12	75.0
Automated identification	1/1	100.0	0/0	0.0	8/60	13.3	1/1	100.0	34/40	85.0	1/1	100.0	5/5	100.0	4/4	100.0	29/61	47.5	1/1	100.0	1/1	100.0	5/12	41.7
Biochemical tests	1/1	100.0	0/0	0.0	8/60	13.3	1/1	100.0	27/40	67.5	0/1	0.0	3/5	60.0	4/4	100.0	37/57	64.9	0/0	0.0	1/1	100.0	9/12	75.0
DNA sequencing	1/1	100.0	0/0	0.0	2/59	3.4	0/1	0.0	12/39	30.8	0/1	0.0	1/5	20.0	1/4	25.0	4/64	6.3	0/1	0.0	0/1	0.0	0/12	0.0
MALDI–TOF–MS	0/0	0.0	0/0	0.0	2/59	3.4	1/1	100.0	16/40	40.0	0/1	0.0	1/5	20.0	4/4	100.0	3/62	4.8	0/1	0.0	1/1	100.0	0/11	0.0
Mounting medium	1/1	100.0	0/0	0.0	11/58	19.0	1/1	100.0	6/34	17.6	0/1	0.0	0/4	0.0	3/3	100.0	7/58	12.1	0/1	0.0	1/1	100.0	3/9	33.3
Available antifungal susceptibility test technologies	1/1	100.0	0/0	0.0	7/60	11.7	1/1	100.0	37/42	88.1	1/1	100.0	5/5	100.0	4/4	100.0	40/66	60.6	1/1	100.0	1/1	100.0	9/12	75.0
Broth microdilution. using CLSI standards	1/1	100.0	0/0	0.0	3/58	5.2	0/1	0.0	18/39	46.2	0/1	0.0	3/4	75.0	3/3	100.0	15/56	26.8	1/1	100.0	1/1	100.0	4/12	33.3
Broth microdilution. using EUCAST standards	1/1	100.0	0/0	0.0	0/58	0.0	0/1	0.0	7/37	18.9	0/1	0.0	0/4	0.0	1/3	33.3	2/54	3.7	0/0	0.0	1/1	100.0	1/11	9.1
Gradient diffusion test	0/0	0.0	0/0	0.0	0/58	0.0	1/1	100.0	12/39	30.8	0/1	0.0	1/4	25.0	3/3	100.0	7/53	13.2	0/0	0.0	1/1	100.0	6/12	50.0
Automated identification	0/0	0.0	0/0	0.0	6/60	10.0	1/1	100.0	32/40	80.0	1/1	100.0	5/5	100.0	2/3	66.7	18/54	33.3	0/0	0.0	1/1	100.0	5/12	41.7
**Serology**	1/1	100.0	0/0	0.0	4/11	36.4	0/0	0.0	29/40	72.5	1/1	100.0	2/5	40.0	3/4	75.0	19/53	35.8	1/1	100.0	1/1	100.0	9/12	75.0
Aspergillus spp.	1/1	100.0	0/0	0.0	2/10	20.0	0/0	0.0	22/40	55.0	0/1	0.0	2/5	40.0	1/4	25.0	13/52	25.0	0/0	0.0	1/1	100.0	8/11	72.7
Onsite	1/1	100.0	0/0	0.0	0/10	0.0	0/0	0.0	14/40	35.0	0/1	0.0	2/5	40.0	1/4	25.0	4/52	7.7	0/0	0.0	1/1	100.0	6/11	54.5
Outsourced	0/1	0.0	0/0	0.0	2/10	20.0	0/0	0.0	8/40	20.0	0/1	0.0	0/5	0.0	0/4	0.0	9/52	17.3	0/0	0.0	0/1	0.0	2/11	18.2
Candida spp.	1/1	100.0	0/0	0.0	3/10	30.0	0/0	0.0	13/40	32.5	1/1	100.0	1/5	20.0	0/4	0.0	10/52	19.2	1/1	100.0	0/1	0.0	2/11	18.2
Onsite	1/1	100.0	0/0	0.0	0/10	0.0	0/0	0.0	7/40	17.5	0/1	0.0	1/5	20.0	0/4	0.0	3/52	5.8	1/1	100.0	0/1	0.0	2/11	18.2
Outsourced	0/1	0.0	0/0	0.0	3/10	30.0	0/0	0.0	6/40	15.0	1/1	100.0	0/5	0.0	0/4	0.0	7/52	13.5	0/1	0.0	0/1	0.0	0/11	0.0
Histoplasma spp.	1/1	100.0	0/0	0.0	4/11	36.4	0/0	0.0	26/40	65.0	0/1	0.0	1/5	20.0	2/4	50.0	17/52	32.7	0/1	0.0	1/1	100.0	9/12	75.0
Onsite	1/1	100.0	0/0	0.0	1/11	9.1	0/0	0.0	14/40	35.0	0/1	0.0	0/5	0.0	2/4	50.0	3/52	5.8	0/1	0.0	1/1	100.0	6/12	50.0
Outsourced	0/1	0.0	0/0	0.0	3/11	27.3	0/0	0.0	12/40	30.0	0/1	0.0	1/5	20.0	0/4	0.0	14/52	26.9	0/1	0.0	0/1	0.0	3/12	25.0
Paracoccidioides spp.	0/0	0.0	0/0	0.0	2/10	20.0	0/0	0.0	6/37	16.2	0/1	0.0	1/5	20.0	3/4	75.0	13/52	25.0	0/1	0.0	1/1	100.0	9/12	75.0
Onsite	0/0	0.0	0/0	0.0	0/10	0.0	0/0	0.0	1/37	2.7	0/1	0.0	0/5	0.0	3/4	75.0	2/52	3.8	0/1	0.0	1/1	100.0	6/12	50.0
Outsourced	0/0	0.0	0/0	0.0	2/10	20.0	0/0	0.0	5/37	13.5	0/1	0.0	1/5	20.0	0/4	0.0	11/52	21.2	0/1	0.0	0/1	0.0	3/12	25.0
**Antigen detection**	1/1	100.0	0/0	0.0	15/17	88.2	1/1	100.0	35/41	85.4	1/1	100.0	5/5	100.0	4/4	100.0	36/62	58.1	0/1	0.0	1/1	100.0	9/12	75.0
Aspergillus spp. GM	1/1	100.0	0/0	0.0	9/13	69.2	1/1	100.0	29/41	70.7	1/1	100.0	3/5	60.0	3/4	75.0	19/62	30.6	0/1	0.0	1/1	100.0	5/11	45.5
Aspergillus spp. GM (ELISA)	1/1	100.0	0/0	0.0	7/12	58.3	0/1	0.0	18/37	48.6	0/1	0.0	1/4	25.0	1/4	25.0	17/55	30.9	0/1	0.0	1/1	100.0	1/11	9.1
Onsite	1/1	100.0	0/0	0.0	2/12	16.7	0/1	0.0	10/37	27.0	0/1	0.0	0/4	0.0	0/4	0.0	7/55	12.7	0/1	0.0	1/1	100.0	0/11	0.0

	GY n=1		HT n=1		HN n=80		JM n=1		MX n=42		NI n=1		PA n=6		PY n=5		PE n=80		TT n=1		UY n=1		VE n=12	
	n	*%*	*n*	*%*	*n*	*%*	*n*	*%*	*n*	*%*	*n*	*%*	*n*	*%*	*n*	*%*	*n*	*%*	*n*	*%*	*n*	*%*	*n*	*%*
Outsourced	0/1	0.0	0/0	0.0	5/12	41.7	0/1	0.0	8/37	21.6	0/1	0.0	1/4	25.0	1/4	25.0	10/55	18.2	0/1	0.0	0/1	0.0	1/11	9.1
Aspergillus spp. GM (LFA)	1/1	100.0	0/0	0.0	5/12	41.7	1/1	100.0	16/39	41.0	0/1	0.0	2/4	50.0	2/3	66.7	10/61	16.4	0/1	0.0	0/1	0.0	5/11	45.5
Onsite	1/1	100.0	0/0	0.0	1/12	8.3	1/1	100.0	11/39	28.2	0/1	0.0	0/4	0.0	2/3	66.7	2/61	3.3	0/1	0.0	0/1	0.0	4/11	36.4
Outsourced	0/1	0.0	0/0	0.0	4/12	33.3	0/1	0.0	5/39	12.8	0/1	0.0	2/4	50.0	0/3	0.0	8/61	13.1	0/1	0.0	0/1	0.0	1/11	9.1
Aspergillus spp. GM (LFD)	1/1	100.0	0/0	0.0	2/9	22.2	0/1	0.0	18/40	45.0	1/1	100.0	3/5	60.0	1/4	25.0	8/52	15.4	0/1	0.0	0/1	0.0	5/11	45.5
Onsite	1/1	100.0	0/0	0.0	1/9	11.1	0/1	0.0	9/40	22.5	0/1	0.0	1/5	20.0	0/4	0.0	2/52	3.8	0/1	0.0	0/1	0.0	4/11	36.4
Outsourced	0/1	0.0	0/0	0.0	1/9	11.1	0/1	0.0	9/40	22.5	1/1	100.0	2/5	40.0	1/4	25.0	6/52	11.5	0/1	0.0	0/1	0.0	1/11	9.1
Candida spp.	1/1	100.0	0/0	0.0	5/11	45.5	0/0	0.0	7/38	18.4	1/1	100.0	1/5	20.0	0/4	0.0	7/61	11.5	0/1	0.0	0/1	0.0	2/11	18.2
Onsite	1/1	100.0	0/0	0.0	2/11	18.2	0/0	0.0	2/38	5.3	0/1	0.0	0/5	0.0	0/4	0.0	0/61	0.0	0/1	0.0	0/1	0.0	1/11	9.1
Outsourced	0/1	0.0	0/0	0.0	3/11	27.3	0/0	0.0	5/38	13.2	1/1	100.0	1/5	20.0	0/4	0.0	7/61	11.5	0/1	0.0	0/1	0.0	1/11	9.1
Cryptococcus spp. GM	1/1	100.0	0/0	0.0	9/14	64.3	1/1	100.0	31/40	77.5	1/1	100.0	5/5	100.0	4/4	100.0	33/61	54.1	0/1	0.0	1/1	100.0	6/12	50.0
Cryptococcus spp. GM (LAT)	1/1	100.0	0/0	0.0	4/12	33.3	0/0	0.0	25/40	62.5	0/1	0.0	3/5	60.0	2/4	50.0	21/51	41.2	0/1	0.0	1/1	100.0	6/12	50.0
Onsite	1/1	100.0	0/0	0.0	2/12	16.7	0/0	0.0	15/40	37.5	0/1	0.0	2/5	40.0	2/4	50.0	14/51	27.5	0/1	0.0	1/1	100.0	5/12	41.7
Outsourced	0/1	0.0	0/0	0.0	2/12	16.7	0/0	0.0	10/40	25.0	0/1	0.0	1/5	20.0	0/4	0.0	7/51	13.7	0/1	0.0	0/1	0.0	1/12	8.3
Cryptococcus spp. GM (LFA)	1/1	100.0	0/0	0.0	9/14	64.3	1/1	100.0	19/37	51.4	1/1	100.0	4/5	80.0	2/4	50.0	19/61	31.1	0/1	0.0	0/1	0.0	2/11	18.2
Onsite	1/1	100.0	0/0	0.0	4/14	28.6	1/1	100.0	13/37	35.1	1/1	100.0	3/5	60.0	2/4	50.0	11/61	18.0	0/1	0.0	0/1	0.0	1/11	9.1
Outsourced	0/1	0.0	0/0	0.0	5/14	35.7	0/1	0.0	6/37	16.2	0/1	0.0	1/5	20.0	0/4	0.0	8/61	13.1	0/1	0.0	0/1	0.0	1/11	9.1
Histoplasma spp.	1/1	100.0	0/0	0.0	10/14	71.4	0/0	0.0	26/40	65.0	1/1	100.0	2/5	40.0	1/4	25.0	21/61	34.4	0/1	0.0	1/1	100.0	3/12	25.0
Onsite	1/1	100.0	0/0	0.0	4/14	28.6	0/0	0.0	10/40	25.0	0/1	0.0	0/5	0.0	1/4	25.0	4/61	6.6	0/1	0.0	0/1	0.0	2/12	16.7
Outsourced	0/1	0.0	0/0	0.0	6/14	42.9	0/0	0.0	16/40	40.0	1/1	100.0	2/5	40.0	0/4	0.0	17/61	27.9	0/1	0.0	1/1	100.0	1/12	8.3
β-D-glucan	1/1	100.0	0/0	0.0	1/11	9.1	1/1	100.0	14/39	35.9	1/1	100.0	2/5	40.0	0/4	0.0	8/61	13.1	0/1	0.0	0/1	0.0	1/11	9.1
Onsite	1/1	100.0	0/0	0.0	0/11	0.0	1/1	100.0	2/39	5.1	0/1	0.0	0/5	0.0	0/4	0.0	0/61	0.0	0/1	0.0	0/1	0.0	0/11	0.0
Outsourced	0/1	0.0	0/0	0.0	1/11	9.1	0/1	0.0	12/39	30.8	1/1	100.0	2/5	40.0	0/4	0.0	8/61	13.1	0/1	0.0	0/1	0.0	1/11	9.1
**Molecular tests**	1/1	100.0	0/0	0.0	2/65	3.1	0/0	0.0	17/40	42.5	0/1	0.0	3/5	60.0	3/4	75.0	13/53	24.5	0/1	0.0	1/1	100.0	1/11	9.1
Aspergillus spp.	1/1	100.0	0/0	0.0	1/8	12.5	0/0	0.0	13/40	32.5	0/1	0.0	1/5	20.0	3/4	75.0	9/51	17.6	0/1	0.0	0/1	0.0	1/11	9.1
Onsite	1/1	100.0	0/0	0.0	1/8	12.5	0/0	0.0	7/40	17.5	0/1	0.0	1/5	20.0	2/4	50.0	2/51	3.9	0/1	0.0	0/1	0.0	0/11	0.0
Outsourced	0/1	0.0	0/0	0.0	0/8	0.0	0/0	0.0	6/40	15.0	0/1	0.0	0/5	0.0	1/4	25.0	7/51	13.7	0/1	0.0	0/1	0.0	1/11	9.1
Candida spp.	1/1	100.0	0/0	0.0	2/9	22.2	0/0	0.0	12/40	30.0	0/1	0.0	3/5	60.0	2/4	50.0	9/51	17.6	0/1	0.0	0/1	0.0	1/11	9.1
Onsite	1/1	100.0	0/0	0.0	1/9	11.1	0/0	0.0	7/40	17.5	0/1	0.0	3/5	60.0	1/4	25.0	1/51	2.0	0/1	0.0	0/1	0.0	0/11	0.0
Outsourced	0/1	0.0	0/0	0.0	1/9	11.1	0/0	0.0	5/40	12.5	0/1	0.0	0/5	0.0	1/4	25.0	8/51	15.7	0/1	0.0	0/1	0.0	1/11	9.1
Pneumocystis jirovecii	1/1	100.0	0/0	0.0	1/9	11.1	0/0	0.0	10/39	25.6	0/1	0.0	0/5	0.0	1/4	25.0	10/53	18.9	0/1	0.0	1/1	100.0	1/11	9.1
Onsite	1/1	100.0	0/0	0.0	0/9	0.0	0/0	0.0	4/39	10.3	0/1	0.0	0/5	0.0	1/4	25.0	0/53	0.0	0/1	0.0	0/1	0.0	0/11	0.0
Outsourced	0/1	0.0	0/0	0.0	1/9	11.1	0/0	0.0	6/39	15.4	0/1	0.0	0/5	0.0	0/4	0.0	10/53	18.9	0/1	0.0	1/1	100.0	1/11	9.1
Mucorales	0/1	0.0	0/0	0.0	0/8	0.0	0/0	0.0	11/37	29.7	0/1	0.0	0/5	0.0	0/3	0.0	7/50	14.0	0/1	0.0	0/1	0.0	1/11	9.1
Onsite	0/1	0.0	0/0	0.0	0/8	0.0	0/0	0.0	6/37	16.2	0/1	0.0	0/5	0.0	0/3	0.0	0/50	0.0	0/1	0.0	0/1	0.0	0/11	0.0
Outsourced	0/1	0.0	0/0	0.0	0/8	0.0	0/0	0.0	5/37	13.5	0/1	0.0	0/5	0.0	0/3	0.0	7/50	14.0	0/1	0.0	0/1	0.0	1/11	9.1
**Imaging procedures**																								
CT	1/1	100.0	0/0	0.0	7/23	30.4	1/1	100.0	33/37	89.2	1/1	100.0	5/5	100.0	2/3	66.7	45/55	81.8	1/1	100.0	1/1	100.0	4/11	36.4
PET CT	0/1	0.0	0/0	0.0	1/23	4.3	0/1	0.0	13/37	35.1	0/1	0.0	0/5	0.0	0/3	0.0	4/55	7.3	0/1	0.0	1/1	100.0	1/11	9.1
MRI	1/1	100.0	0/0	0.0	5/23	21.7	1/1	100.0	30/37	81.1	1/1	100.0	3/5	60.0	2/3	66.7	16/55	29.1	1/1	100.0	1/1	100.0	3/11	27.3

	GY n=1		HT n=1		HN n=80		JM n=1		MX n=42		NI n=1		PA n=6		PY n=5		PE n=80		TT n=1		UY n=1		VE n=12	
	n	*%*	*n*	*%*	*n*	*%*	*n*	*%*	*n*	*%*	*n*	*%*	*n*	*%*	*n*	*%*	*n*	*%*	*n*	*%*	*n*	*%*	*n*	*%*
PET MRI	0/1	0.0	0/0	0.0	0/23	0.0	0/1	0.0	4/37	10.8	0/1	0.0	0/5	0.0	0/3	0.0	3/55	5.5	0/1	0.0	0/1	0.0	1/11	9.1
Ultrasound	1/1	100.0	0/0	0.0	12/23	52.2	1/1	100.0	32/37	86.5	1/1	100.0	5/5	100.0	3/3	100.0	49/55	89.1	1/1	100.0	1/1	100.0	7/11	63.6
X ray	1/1	100.0	0/0	0.0	12/23	52.2	1/1	100.0	34/37	91.9	1/1	100.0	4/5	80.0	1/3	33.3	43/55	78.2	1/1	100.0	1/1	100.0	6/11	54.5
**Surgery**	1/1	100.0	0/0	0.0	6/16	37.5	1/1	100.0	31/36	86.1	1/1	100.0	4/5	80.0	1/2	50.0	21/38	55.3	1/1	100.0	1/1	100.0	3/9	33.3
**Systemic antifungals**																								
Amphotericin B	1/1	100.0	0/0	0.0	11/24	45.8	1/1	100.0	38/41	92.7	1/1	100.0	6/6	100.0	3/3	100.0	58/67	86.6	1/1	100.0	1/1	100.0	9/10	90.0
Deoxycholate	1/1	100.0	0/0	0.0	10/24	41.7	1/1	100.0	32/39	82.1	1/1	100.0	6/6	100.0	3/3	100.0	55/67	82.1	1/1	100.0	1/1	100.0	7/8	87.5
Lipid-based formulations	1/1	100.0	0/0	0.0	7/24	29.2	0/1	0.0	31/40	77.5	0/1	0.0	0/6	0.0	3/3	100.0	12/67	17.9	1/1	100.0	1/1	100.0	4/10	40.0
Lipidic complex	1/1	100.0	0/0	0.0	2/23	8.7	0/1	0.0	6/39	15.4	0/1	0.0	0/6	0.0	1/3	33.3	3/67	4.5	1/1	100.0	0/1	0.0	2/9	22.2
Liposomal	1/1	100.0	0/0	0.0	7/24	29.2	0/1	0.0	30/40	75.0	0/1	0.0	0/6	0.0	2/3	66.7	11/67	16.4	0/0	0.0	1/1	100.0	3/9	33.3
Echinocandins	0/1	0.0	0/0	0.0	8/23	34.8	1/1	100.0	35/41	85.4	1/1	100.0	5/6	83.3	1/3	33.3	29/69	42.0	1/1	100.0	1/1	100.0	8/10	80.0
Anidulafungin	0/1	0.0	0/0	0.0	6/23	26.1	0/1	0.0	21/41	51.2	1/1	100.0	2/6	33.3	1/3	33.3	9/67	13.4	0/0	0.0	0/1	0.0	2/9	22.2
Caspofungin	0/1	0.0	0/0	0.0	2/23	8.7	1/1	100.0	26/39	66.7	1/1	100.0	5/6	83.3	0/3	0.0	24/68	35.3	1/1	100.0	1/1	100.0	7/9	77.8
Micafungin	0/1	0.0	0/0	0.0	3/23	13.0	1/1	100.0	4/38	10.5	0/1	0.0	0/6	0.0	0/3	0.0	1/68	1.5	0/0	0.0	0/1	0.0	0/8	0.0
Triazoles	1/1	100.0	1/1	100.0	20/24	83.3	1/1	100.0	42/42	100.0	1/1	100.0	6/6	100.0	3/3	100.0	67/69	97.1	1/1	100.0	1/1	100.0	10/10	100.0
Fluconazole	1/1	100.0	1/1	100.0	19/24	79.2	1/1	100.0	42/42	100.0	1/1	100.0	6/6	100.0	3/3	100.0	66/69	95.7	1/1	100.0	1/1	100.0	10/10	100.0
Mould-active triazoles	1/1	100.0	0/0	0.0	14/24	58.3	1/1	100.0	40/41	97.6	1/1	100.0	6/6	100.0	3/3	100.0	59/69	85.5	1/1	100.0	1/1	100.0	10/10	100.0
Itraconazole	1/1	100.0	0/0	0.0	6/24	25.0	1/1	100.0	28/40	70.0	1/1	100.0	6/6	100.0	3/3	100.0	50/68	73.5	1/1	100.0	1/1	100.0	7/9	77.8
Isavuconazole	0/1	0.0	0/0	0.0	8/23	34.8	0/1	0.0	18/40	45.0	0/1	0.0	0/6	0.0	0/3	0.0	14/67	20.9	0/0	0.0	1/1	100.0	1/8	12.5
Posaconazole	0/1	0.0	0/0	0.0	0/23	0.0	0/1	0.0	14/39	35.9	0/1	0.0	0/6	0.0	0/3	0.0	15/67	22.4	1/1	100.0	0/1	0.0	1/8	12.5
Voriconazole	0/1	0.0	0/0	0.0	6/23	26.1	1/1	100.0	34/40	85.0	1/1	100.0	5/6	83.3	1/3	33.3	27/66	40.9	1/1	100.0	1/1	100.0	9/10	90.0
Flucytosine	1/1	100.0	0/0	0.0	0/23	0.0	0/1	0.0	0/38	0.0	0/1	0.0	0/6	0.0	0/3	0.0	1/68	1.5	1/1	100.0	0/1	0.0	1/8	12.5
Terbinafine	0/1	0.0	0/0	0.0	5/22	22.7	1/1	100.0	16/39	41.0	0/1	0.0	1/6	16.7	0/3	0.0	25/63	39.7	0/0	0.0	1/1	100.0	8/10	80.0
**Therapeutic drug monitoring**	1/1	100.0	0/0	0.0	7/18	38.9	0/1	0.0	14/36	38.9	0/1	0.0	0/5	0.0	0/4	0.0	5/57	8.8	0/0	0.0	1/1	100.0	1/10	10.0
Flucytosine	1/1	100.0	0/0	0.0	0/15	0.0	0/1	0.0	0/33	0.0	0/1	0.0	0/5	0.0	0/4	0.0	0/56	0.0	0/0	0.0	0/1	0.0	0/10	0.0
Onsite	1/1	100.0	0/0	0.0	0/15	0.0	0/1	0.0	0/33	0.0	0/1	0.0	0/5	0.0	0/4	0.0	0/56	0.0	0/0	0.0	0/1	0.0	0/10	0.0
Outsourced	0/1	0.0	0/0	0.0	0/15	0.0	0/1	0.0	0/33	0.0	0/1	0.0	0/5	0.0	0/4	0.0	0/56	0.0	0/0	0.0	0/1	0.0	0/10	0.0
Isavuconazole	0/1	0.0	0/0	0.0	3/17	17.6	0/1	0.0	3/30	10.0	0/1	0.0	0/5	0.0	0/2	0.0	1/54	1.9	0/0	0.0	0/1	0.0	0/5	0.0
Onsite	0/1	0.0	0/0	0.0	0/17	0.0	0/1	0.0	0/30	0.0	0/1	0.0	0/5	0.0	0/2	0.0	1/54	1.9	0/0	0.0	0/1	0.0	0/5	0.0
Outsourced	0/1	0.0	0/0	0.0	3/17	17.6	0/1	0.0	3/30	10.0	0/1	0.0	0/5	0.0	0/2	0.0	0/54	0.0	0/0	0.0	0/1	0.0	0/5	0.0
Itraconazole	1/1	100.0	0/0	0.0	3/13	23.1	0/1	0.0	11/34	32.4	0/1	0.0	0/5	0.0	0/2	0.0	4/54	7.4	0/0	0.0	0/1	0.0	1/8	12.5
Onsite	1/1	100.0	0/0	0.0	3/13	23.1	0/1	0.0	7/34	20.6	0/1	0.0	0/5	0.0	0/2	0.0	3/54	5.6	0/0	0.0	0/1	0.0	1/8	12.5
Outsourced	0/1	0.0	0/0	0.0	0/13	0.0	0/1	0.0	4/34	11.8	0/1	0.0	0/5	0.0	0/2	0.0	1/54	1.9	0/0	0.0	0/1	0.0	0/8	0.0
Posaconazole	0/1	0.0	0/0	0.0	0/14	0.0	0/1	0.0	7/35	20.0	0/1	0.0	0/5	0.0	0/2	0.0	2/53	3.8	0/0	0.0	0/1	0.0	0/7	0.0
Onsite	0/1	0.0	0/0	0.0	0/14	0.0	0/1	0.0	2/35	5.7	0/1	0.0	0/5	0.0	0/2	0.0	2/53	3.8	0/0	0.0	0/1	0.0	0/7	0.0
Outsourced	0/1	0.0	0/0	0.0	0/14	0.0	0/1	0.0	5/35	14.3	0/1	0.0	0/5	0.0	0/2	0.0	0/53	0.0	0/0	0.0	0/1	0.0	0/7	0.0
Voriconazole	1/1	100.0	0/0	0.0	3/14	21.4	0/1	0.0	11/35	31.4	0/1	0.0	0/5	0.0	0/2	0.0	3/53	5.7	0/0	0.0	1/1	100.0	1/9	11.1
Onsite	1/1	100.0	0/0	0.0	3/14	21.4	0/1	0.0	8/35	22.9	0/1	0.0	0/5	0.0	0/2	0.0	3/53	5.7	0/0	0.0	1/1	100.0	1/9	11.1
Outsourced	0/1	0.0	0/0	0.0	0/14	0.0	0/1	0.0	3/35	8.6	0/1	0.0	0/5	0.0	0/2	0.0	0/53	0.0	0/0	0.0	0/1	0.0	0/9	0.0

CLSI, Clinical and Laboratory Standards Institute; **CT**, computed tomography; **DNA**, deoxyribonucleic acid; **ELISA**, enzyme-linked immunosorbent assay; **EUCAST**, European Committee on Antimicrobial Susceptibility Testing; **GM**, galactomannan; **GY**, Guyana; **HIV**, human immunodeficiency virus; **HN**, Honduras; **HT**, Haiti; **HSCT**, hematopoietic stem cell transplantation; **IFD**, invasive fungal disease; **JM**, Jamaica; **KOH**, potassium hydroxide; **LAT**, latex agglutination test; **LFA**, lateral flow assay; **LFD**, lateral flow device; **MALDI–TOF–MS**, matrix-assisted laser desorption/ionization time-of-flight mass spectrometry; **MX**, Mexico; **MRI**, magnetic resonance imaging; **n**, number (sample size); **NI**, Nicaragua; **PA**, Panama; **p**, probability; **PET**, positron emission tomography; **PE**, Peru; **PY**, Paraguay; **SOT**, solid organ transplantation; **spp.**, species; **TT**, Trinidad and Tobago; **UY**, Uruguay; **VE**, Venezuela
