# Supplementary material for: Fungal diagnostics and antifungal drug access in Latin America and the Caribbean: an ESCMID EFISG multinational survey
Source: Nat Commun. 2026 Jul 28;17:7552. doi: 10.1038/s41467-026-73165-2 (PMC13415557; doi:10.1038/s41467-026-73165-2)
Supplement: Supplementary file 2 — Reporting Summary [file 41467_2026_73165_MOESM2_ESM.pdf]

nature portfolio

Corresponding author(s): NCOMMS-25-102535-T

Last updated by author(s): Mar 4, 2026

Reporting Summary

Nature Portfolio wishes to improve the reproducibility of the work that we publish. This form provides structure for consistency and transparency in reporting. For further information on Nature Portfolio policies, see our Editorial Policies and the Editorial Policy Checklist.

Statistics

For all statistical analyses, confirm that the following items are present in the figure legend, table legend, main text, or Methods section.

n/a	Confirmed	
☐	☒	The exact sample size (*n*) for each experimental group/condition, given as a discrete number and unit of measurement
☒	☐	A statement on whether measurements were taken from distinct samples or whether the same sample was measured repeatedly
☐	☒	The statistical test(s) used AND whether they are one- or two-sided<br>*Only common tests should be described solely by name; describe more complex techniques in the Methods section.*
☒	☐	A description of all covariates tested
☒	☐	A description of any assumptions or corrections, such as tests of normality and adjustment for multiple comparisons
☐	☒	A full description of the statistical parameters including central tendency (e.g. means) or other basic estimates (e.g. regression coefficient) AND variation (e.g. standard deviation) or associated estimates of uncertainty (e.g. confidence intervals)
☒	☐	For null hypothesis testing, the test statistic (e.g. *F*, *t*, *r*) with confidence intervals, effect sizes, degrees of freedom and *P* value noted<br>*Give P values as exact values whenever suitable.*
☒	☐	For Bayesian analysis, information on the choice of priors and Markov chain Monte Carlo settings
☒	☐	For hierarchical and complex designs, identification of the appropriate level for tests and full reporting of outcomes
☒	☐	Estimates of effect sizes (e.g. Cohen's *d*, Pearson's *r*), indicating how they were calculated

Our web collection on statistics for biologists contains articles on many of the points above.

Software and code

Policy information about availability of computer code

Data collection	Data were collected using a secure web-based platform (EFS Survey, TIVIAN GmbH, Cologne, Germany).
Data analysis	Statistical analyses were performed using SPSS (IBM Corp., Armonk, NY, USA).

For manuscripts utilizing custom algorithms or software that are central to the research but not yet described in published literature, software must be made available to editors and reviewers. We strongly encourage code deposition in a community repository (e.g. GitHub). See the Nature Portfolio guidelines for submitting code & software for further information.

Data

Policy information about availability of data

All manuscripts must include a data availability statement. This statement should provide the following information, where applicable:

- Accession codes, unique identifiers, or web links for publicly available datasets
- A description of any restrictions on data availability
- For clinical datasets or third party data, please ensure that the statement adheres to our policy

All aggregated data supporting the findings of this study are included in the manuscript and Supplementary Information.Anonymized institutional-level survey data are available from the corresponding author upon reasonable request.No third-party or restricted clinical datasets were used.

Research involving human participants, their data, or biological material

Policy information about studies with human participants or human data. See also policy information about sex, gender (identity/presentation), and sexual orientation and race, ethnicity and racism.

Reporting on sex and gender	The study assessed institutional diagnostic and therapeutic capacity and did not collect individual-level patient data; therefore, sex and gender variables were not applicable.
Reporting on race, ethnicity, or other socially relevant groupings	No individual-level demographic data were collected. The study focused on institutional infrastructure and access to diagnostics and treatment.
Population characteristics	Participating units were hospitals, laboratories, and reference centers involved in invasive fungal disease diagnosis or management across 23 countries in Latin America and the Caribbean.
Recruitment	Institutions were recruited via national focal points through professional networks and scientific societies. Participation was voluntary. Up to five reminders were sent to maximize participation.
Ethics oversight	Ethical approval was obtained from the Institutional Review Board (IRB) of the Brazilian institution for the Brazilian authors, as required.The study did not involve human subjects, patient-level data, or biological samples. Institutional participation required electronic informed consent.

Note that full information on the approval of the study protocol must also be provided in the manuscript.

Field-specific reporting

Please select the one below that is the best fit for your research. If you are not sure, read the appropriate sections before making your selection.

☐ Life sciences ☒ Behavioural & social sciences ☐ Ecological, evolutionary & environmental sciences

For a reference copy of the document with all sections, see nature.com/documents/nr-reporting-summary-flat.pdf

Behavioural & social sciences study design

All studies must disclose on these points even when the disclosure is negative.

Study description	This study is a cross-sectional, multinational, online survey assessing diagnostic capacity, antifungal availability, and therapeutic drug monitoring (TDM) for invasive fungal diseases (IFD) across Latin America and the Caribbean (LAC).
Research sample	A total of 619 institutions (hospitals, clinical laboratories, and reference centres) from 23 countries in Latin America and the Caribbean participated. Eligible institutions were those involved in IFD diagnosis and/or management.
Sampling strategy	Institutions were recruited via national focal points identified through professional networks, scientific societies, and regional organizations (including PAHO). The survey was distributed via a secure web-based platform. Participation was voluntary. Up to five reminders were sent to maximize response rates.
Data collection	Data were collected using a secure web-based survey platform (EFS Survey, TIVIAN GmbH, Cologne, Germany).
Timing	Data were collected between April 2023 and May 2025.
Data exclusions	Incomplete responses were included in analyses when relevant data were available. No additional exclusion criteria were applied.
Non-participation	Participation was voluntary. Institutions that did not respond were not included in the analysis.
Randomization	Randomization was not part of the study design, as this was an observational survey.

Reporting for specific materials, systems and methods

We require information from authors about some types of materials, experimental systems and methods used in many studies. Here, indicate whether each material, system or method listed is relevant to your study. If you are not sure if a list item applies to your research, read the appropriate section before selecting a response.

Materials & experimental systems

n/a	Involved in the study
☒	☐ Antibodies
☒	☐ Eukaryotic cell lines
☒	☐ Palaeontology and archaeology
☒	☐ Animals and other organisms
☐	☒ Clinical data
☒	☐ Dual use research of concern
☒	☐ Plants

Methods

n/a	Involved in the study
☒	☐ ChIP-seq
☒	☐ Flow cytometry
☒	☐ MRI-based neuroimaging

Clinical data

Policy information about clinical studies

All manuscripts should comply with the ICMJE guidelines for publication of clinical research and a completed CONSORT checklist must be included with all submissions.

Clinical trial registration	This study did not involve a clinical trial. The research consisted of an institutional-level cross-sectional survey assessing diagnostic and therapeutic capacity for invasive fungal diseases.
Study protocol	The study protocol consisted of a structured multinational survey designed to assess availability of mycological diagnostics, antifungal therapies, and therapeutic drug monitoring across institutions in Latin America and the Caribbean. The survey design, eligibility criteria, and analysis plan are described in the Methods section of the manuscript.
Data collection	Data were collected using a structured online questionnaire completed by representatives of participating institutions (e.g., clinicians, microbiologists, or laboratory personnel). The survey captured institutional-level information on diagnostic infrastructure, availability of antifungal agents, and clinical services related to invasive fungal disease management.
Outcomes	The primary outcomes were institutional availability and access to diagnostic methods for invasive fungal diseases, antifungal treatment options, and therapeutic drug monitoring. Secondary outcomes included comparisons of diagnostic and treatment capacities across institutional characteristics and national economic indicators.

Plants

Seed stocks	Not applicable
Novel plant genotypes	Not applicable
Authentication	Not applicable
