# Supplementary material for: Fungal diagnostics and antifungal drug access in Latin America and the Caribbean: an ESCMID EFISG multinational survey
Source: Nat Commun. 2026 Jul 28;17:7552. doi: 10.1038/s41467-026-73165-2 (PMC13415557; doi:10.1038/s41467-026-73165-2)
Supplement: Supplementary file 3 — Transparent Peer Review file [file 41467_2026_73165_MOESM3_ESM.pdf]

nature portfolio

Peer Review File

Fungal diagnostics and antifungal drug access in Latin America and the Caribbean: An ESCMID EFISG Multinational Survey

Corresponding Author: Dr Jon Salmanton-García

This file contains all reviewer reports in order by version, followed by all author rebuttals in order by version.

Version 0:

Reviewer comments:

Reviewer #1

(Remarks to the Author)
This manuscript summarised the findings of a large scale survey that was performed across Latin America and the Caribbean on the diagnostic and treatment capabilities of institutions. This work presents a real large scale effort to collect data from many countries that are generally underrepresented when it comes to large scale studies. This data will be crucial for advancing diagnostic and treatment for fungal diseases and provides a clear and nice summary of the current landscape across Latin America and the Caribbean.
While this shows fascinating summary data, I do believe there is more scrutiny of the data to be performed as the large proportion of centers in Colombia and Brazil can bias summary data. As mentioned in the discussion section: heterogeneity between centers and countries were found, but this is not really analysed which I believe it should.
Comments:
- L50-51 perhaps distinguish here that Candida auris, Crypto and Aspergillus fumigatus are critical and Histoplasma is on the High list.
- Table 1: I would like to see all countries percentage plotted as a distribution for each fungal pathogen – this would give insight in what countries are outliers and perceive different pathogens in a different way. More country specific guidance can then be eluded to. Same goes for the incidence actually and most other data.
- L123 I would not consider KOH a stain, it is way to prepare.
- L121-132 Was there a difference in type of institution? Analysing this further where the bottlenecks are would be great in terms of setting out the next few years of diagnostic improvement focus.
- The same goes for the diagnostic capacities analyses across countries, while summary data is really useful this might mask differences in countries and type of institutes. It would be ideal to show distributions to highlight which countries focus on certain areas of improvement.
- For the antifungal analyses, a similar comment, are there countries lacking in certain classes of antifungals or is this difference in within-country institute. Critical to know these things.
- As GDP is a continuum why were these three categories chosen? Is a linear model and therefore statistics not more powerful?
- Section from L189 is very useful and a great type of analysis. The same goes for section L214.
- Fig 1B, C, D and E are of low quality.

Reviewer #2

(Remarks to the Author)
Authors have conducted a large study on the diagnostic and therapeutic capacity in LAC. There were studies from Asia, Africa and Europe, however there was no consolidated data from LAC region.
There are only few comments
Authors can compare the LAC results with other regions little bit in detail
Only few Caribbean countries participated in the survey, however title says entire Caribbean. This can be highlighted in the limitations.
Was there an attempt made to distribute survey to Dutch speaking islands? This is also one of the limitation

Line 113 and table 1 - it is mentioned as 617 participants, is it correct?
Line 42- Sporotrichosis is not systemic mycoses agent
Any data collected on C. auris will be of interest

Reviewer #3

(Remarks to the Author)
1. L108. The countries only depict part of the story. What level of hospital and what size population do they serve? One would not expect a small local hospital to have a fully equipped mycology service, but it would be expected in teaching and major referral hospitals. This is especially true for imaging. These details, if available could be inserted in a new section of results below the section on results by GDP. Transplant is a proxy for specialised/teaching centres, but what about chronic chest disease/TB and intensive care?
2. L108. Also list countries with populations> ?250,000 without any data (ie Puerto Rica) and those with limited data (ie Suriname, Haiti, Jamaica and Nicaragua, El Salvador, French Guiana (note spelling error L495), Guyana, Trinidad and Tobago, and Uruguay).
3. L126. This sentence should be separated out into standard fungal culture (ie respiratory, pus, skin, urine etc) and blood culture. Lack of blood culture is a major problem for AMR control as well as fungal disease. Later (L170) the authors refer to fungal-specific blood culture. This needs clarity generally in the manuscript – was the Q about all blood culture or only specialised B/C? It also needs emphasis in the discussion.
4. L131. An additional sentence is required separating out yeast (Candida) and Aspergillus AFST.
5. L136. Does the Antigen in this sentence refer to Histoplasma? The following lines are unclear about Aspergillus – Antigen, IgG or IgE? (needs clarity in Table S1 as well). Insert Aspergillus IgG and Aspergillus IgE if not here as they are critical for diagnosing chronic and allergic forms of aspergillosis
6. Can the authors make any comment about relative access in private facilities vs public facilities?
7. Supplementary Table 1. Put in bold (or highlight) tests with <50% availability. It is very dense and difficult to read as presented.
8. In which LAC countries is healthcare completely private, completely public or a hybrid? The costs of diagnosis and treatment need to be addressed in the discussion. In LMIC this is a major issue and the authors skip over this.

9. No mention is made of sporotrichosis (other than L42). This problem is a major issue in South America.
10. No mention is made of fungal keratitis, which is moderately common and requires samples to be taken and inoculated in clinic by the clinician. Topical natamycin is a WHO Essential Medicine. The authors focus on invasive fungal disease, but blinding infections are a problem too, and the diagnostics are similar.
11. There are a large proportion of self citations. Others work should be cited. Examples might include: reference to LAC paracoccidioidomycosis guidelines, PAHO Histoplasmosis guidelines, Asia survey of diagnostics (not by these authors), ATS guidelines on transplant-associated infections and others. This should not be a paper with so many self-citations from Koln.

Version 1:

Reviewer comments:

Reviewer #1

(Remarks to the Author)
I appreciate the extra work that has been put in to address my comments and feedback and the additional figure adds weight to the findings.

This reviewer still believes the analysis can be further improved to allow better understanding of differences between countries, however I appreciate this could be another whole manuscript, therefore the manuscript as presented is thorough enough.

This reviewer also disagrees with the other reviewer that there are too many self-citations as much of the work that relates has been put out by the authors, and therefore are relevant to this work.

Open Access This Peer Review File is licensed under a Creative Commons Attribution 4.0 International License, which permits use, sharing, adaptation, distribution and reproduction in any medium or format, as long as you give appropriate credit to the original author(s) and the source, provide a link to the Creative Commons license, and indicate if changes were made.
In cases where reviewers are anonymous, credit should be given to 'Anonymous Referee' and the source.
The images or other third party material in this Peer Review File are included in the article's Creative Commons license, unless indicated otherwise in a credit line to the material. If material is not included in the article's Creative Commons license and your intended use is not permitted by statutory regulation or exceeds the permitted use, you will need to obtain permission directly from the copyright holder.
To view a copy of this license, visit https://creativecommons.org/licenses/by/4.0/

Dear Reviewers,

Following the editorial decision, please find enclosed the revised version of our paper NCOMMS-25-102535-T entitled 'Updated survey on fungal diagnostics and antifungal drug access in Latin America and the Caribbean: A multinational EFISG analysis' that we revised according to the reviewers' helpful suggestions. We appreciated the positive comments, which significantly improved the quality of our paper.

We read with great attention all the stimulating suggestions and accordingly we modified our manuscript, following each point raised and highlighting with track changes the corrections in the text.

**Reviewer #1**

This manuscript summarised the findings of a large scale survey that was performed across Latin America and the Caribbean on the diagnostic and treatment capabilities of institutions. This work presents a real large scale effort to collect data from many countries that are generally underrepresented when it comes to large scale studies. This data will be crucial for advancing diagnostic and treatment for fungal diseases and provides a clear and nice summary of the current landscape across Latin America and the Caribbean.
Answer: We sincerely thank the reviewer for this positive and encouraging assessment. We are particularly grateful for recognizing the value of our effort to collect comprehensive data across Latin America and the Caribbean, regions that are historically underrepresented in large-scale surveys. Your acknowledgment reinforces the relevance of our work in guiding future improvements in diagnostic and therapeutic capacities for invasive fungal diseases across these diverse healthcare settings.

While this shows fascinating summary data, I do believe there is more scrutiny of the data to be performed as the large proportion of centers in Colombia and Brazil can bias summary data. As mentioned in the discussion section: heterogeneity between centers and countries were found, but this is not really analysed which I believe it should.
Answer: We fully appreciate this insightful comment. Indeed, we acknowledge that the higher representation of centers from Colombia and Brazil could influence aggregate regional summaries. To address this, we performed stratified analyses by GDP per capita, transplant activity, and HIV care status. These analyses revealed significant heterogeneity across economic strata and institutional complexity ($p<0.001$), demonstrating that structural and resource-related differences, rather than solely country origin, predominantly drive variability in diagnostic and treatment capacity. While additional country-level sensitivity analyses are in progress, the primary goal of this study remains to provide an overarching characterization of the region, laying the foundation for more detailed future investigations.

Comments:
- L50-51 perhaps distinguish here that *Candida auris*, *Crypto* and *Aspergillus fumigatus* are critical and *Histoplasma* is on the High list.
Answer: We appreciate the reviewer's suggestion to clarify pathogen prioritization. The manuscript has been revised to explicitly indicate that *Candida auris*, *Cryptococcus*, and *Aspergillus fumigatus* are classified as critical priority pathogens, whereas Histoplasma is considered high priority. This distinction has been integrated into the relevant sections for enhanced clarity.

- Table 1: I would like to see all countries percentage plotted as a distribution for each fungal pathogen – this would give insight in what countries are outliers and perceive different pathogens in a different way. More country specific guidance can then be eluded to. Same goes for the incidence actually and most other data.
Answer: We agree that country-level distributions provide valuable insight into outliers and variations in pathogen perception. Accordingly, we have added a new figure in the appendix showing country-specific distributions for key fungal pathogens. Additionally, Appendix Table 1 now

provides disaggregated country-level data for all reported variables. These additions allow readers to appreciate the heterogeneity while preserving the manuscript's focus on regional characterization.

- L123 I would not consider KOH a stain, it is way to prepare.
Answer: We concur that KOH should be described as a preparation method rather than a staining technique. The text has been corrected accordingly.

- L121-132 Was there a difference in type of institution? Analysing this further where the bottlenecks are would be great in terms of setting out the next few years of diagnostic improvement focus.
Answer: We are grateful for this suggestion. While stratified analysis by institution type could provide valuable insights, such analyses are limited by the heterogeneity of healthcare systems and insufficient granularity in our dataset regarding institutional characteristics. We have emphasized this limitation in the manuscript and hope that future studies will specifically address structural bottlenecks in diagnostic capacity.

- The same goes for the diagnostic capacities analyses across countries, while summary data is really useful this might mask differences in countries and type of institutes. It would be ideal to show distributions to highlight which countries focus on certain areas of improvement.
Answer: We acknowledge that more granular country- and institution-specific analyses could be informative. The current manuscript focuses on a regional overview, while dedicated country-specific analyses are underway and will be reported separately.

- For the antifungal analyses, a similar comment, are there countries lacking in certain classes of antifungals or is this difference in within-country institute. Critical to know these things.
Answer: We appreciate the recommendation. In this manuscript, we present an overarching view of antifungal access in the region. Country-specific distributions are now included in the appendix, with more detailed analyses forthcoming in separate publications.

- As GDP is a continuum why were these three categories chosen? Is a linear model and therefore statistics not more powerful?
Answer: While GDP is a continuous variable, we employed categorical stratification consistent with prior publications from this initiative to facilitate comparison across studies and macroeconomic interpretation.

- Section from L189 is very useful and a great type of analysis. The same goes for section L214.
Answer: We very much appreciate the greetings.

- Fig 1B, C, D and E are of low quality.
Answer: We have improved the resolution and visual clarity of these panels to ensure readability and interpretability.

Reviewer #2

Authors have conducted a large study on the diagnostic and therapeutic capacity in LAC. There were studies from Asia, Africa and Europe, however there was no consolidated data from LAC region. There are only few comments

Authors can compare the LAC results with other regions little bit in detail
Answer: We thank the reviewer for this suggestion. We have included in the detailed comparison of the LAC results with findings from other regions, highlighting similarities and differences and offering possible explanations for the observed patterns.

Only few Caribbean countries participated in the survey, however title says entire Caribbean. This can be highlighted in the limitations.

Answer: We agree with the reviewer. Caribbean representation was limited and we have now explicitly acknowledged this as a limitation and clarified that findings may not fully represent less populated Caribbean territories: “Participation from Caribbean islandic countries was limited, primarily affecting smaller island states with relatively low populations. In contrast, larger Caribbean and continental territories, including Mexico, Central America, Colombia, Venezuela, and Guyana, were well represented. Therefore, while the survey provides a robust overview of Latin America and much of the Caribbean, findings may not fully capture diagnostic and therapeutic capacities in the smaller island nations with distinct structural constraints.”.

Was there an attempt made to distribute survey to Dutch speaking islands? This is also one of the limitation
Answer: All the sovereign countries and dependent territories from the region were approached.

Line 113 and table 1 - it is mentioned as 617 participants, is it correct?
Answer: Yes. A total of 619 institutions completed the survey; however, 617 responded to the specific question on self-perceived IFD incidence. This clarification has been incorporated into the Results section.

Line 42- Sporotrichosis is not systemic mycoses agent
Answer: We agree with the reviewer. Sporotrichosis is not a systemic mycosis, and the text has been corrected accordingly.

Any data collected on *C. auris* will be of interest
Answer: We thank the reviewer for this suggestion. The survey was not designed to capture pathogen-specific epidemiological information, and consequently *Candidozyma auris* was not evaluated as a standalone variable. However, the study specifically assessed access to antifungal susceptibility testing and availability of antifungal agents, which are central to the management of drug-resistant *Candida* infections, including *C. auris*. The gaps identified therefore provide relevant insight into regional preparedness for this WHO critical-priority pathogen.

Reviewer #3

1. L108. The countries only depict part of the story. What level of hospital and what size population do they serve? One would not expect a small local hospital to have a fully equipped mycology service, but it would be expected in teaching and major referral hospitals. This is especially true for imaging. These details, if available could be inserted in a new section of results below the section on results by GDP. Transplant is a proxy for specialised/teaching centres, but what about chronic chest disease/TB and intensive care?
Answer: We thank the reviewer for this thoughtful comment. The survey collected detailed data on imaging availability and institutional diagnostic capacity. However, it did not systematically capture hospital level classification, catchment population size, tuberculosis-related services, or ICU-specific infrastructure. Therefore, no conclusions can be drawn regarding these structural service characteristics. Although additional stratified analyses could in principle be explored, we believe that the analyses presented are the most appropriate and aligned with the predefined objectives of this study, and we respectfully propose to retain the current structure as it best reflects the intended scope of the manuscript.

2. L108. Also list countries with populations> ?250,000 without any data (ie Puerto Rica) and those with limited data (ie Suriname, Haiti, Jamaica and Nicaragua, El Salvador, French Guiana (note spelling error L495), Guyana, Trinidad and Tobago, and Uruguay).
Answer: We thank the reviewer for this important observation. Despite extensive regional dissemination of the survey, participation across LAC was heterogeneous, likely reflecting

differences in engagement of local professional networks and institutional participation across countries. Such variability is inherent to large voluntary multicountry initiatives and has been carefully considered when interpreting the findings and their regional generalisability.

3. L126. This sentence should be separated out into standard fungal culture (ie respiratory, pus, skin, urine etc) and blood culture. Lack of blood culture is a major problem for AMR control as well as fungal disease. Later (L170) the authors refer to fungal-specific blood culture. This needs clarity generally in the manuscript – was the Q about all blood culture or only specialised B/C? It also needs emphasis in the discussion.
Answer: We thank the reviewer for this comment. We have removed mentions to blood cultures in order to make more understandable the text and simple.

4. L131. An additional sentence is required separating out yeast (*Candida*) and *Aspergillus* AFST.
Answer: Adapted.

5. L136. Does the Antigen in this sentence refer to *Histoplasma*? The following lines are unclear about *Aspergillus* – Antigen, IgG or IgE? (needs clarity in Table S1 as well). Insert *Aspergillus* IgG and *Aspergillus* IgE if not here as they are critical for diagnosing chronic and allergic forms of aspergillosis
Answer: In the survey, "serology" referred broadly to antibody-based assays without specification of immunoglobulin class. Antigen detection comprised circulating fungal antigen assays, including Histoplasma antigen and Aspergillus galactomannan. Chronic pulmonary and allergic forms of aspergillosis are not classified as invasive fungal diseases; therefore, diagnostic markers specific to these non-invasive entities (e.g., Aspergillus IgG or IgE) were not separately captured, as the survey focused exclusively on invasive fungal diseases.

6. Can the authors make any comment about relative access in private facilities vs public facilities?
Answer: We thank the reviewer for this important point. The survey did not systematically differentiate between public and private healthcare institutions, and therefore no stratified analysis by funding model can be provided. Institutional comparisons were limited to predefined structural variables, including GDP stratification and transplant or HIV care activity.

7. Supplementary Table 1. Put in bold (or highlight) tests with <50% availability. It is very dense and difficult to read as presented.
Answer: We sincerely thank the reviewer for this helpful observation. We fully appreciate that highlighting tests with <50% availability could enhance readability. In accordance with the journal's formatting requirements and considering that no other reviewers raised similar points regarding the supplementary tables, we have retained the current presentation. We believe that the detailed information provided allows readers to interpret the data comprehensively, while maintaining consistency with the journal's style.

8. In which LAC countries is healthcare completely private, completely public or a hybrid? The costs of diagnosis and treatment need to be addressed in the discussion. In LMIC this is a major issue and the authors skip over this.
Answer: The survey was not designed to classify healthcare systems at the country level as fully public, fully private, or hybrid; therefore, no formal categorization can be provided. Across LAC, healthcare delivery models are predominantly mixed, with varying degrees of public–private integration. Cost-related variables were not captured in the survey instrument. Nonetheless, financing frameworks, procurement structures, and direct out-of-pocket health payments are likely to substantially influence access to fungal diagnostics and antifungal therapies, particularly in lower- and middle-income settings.

9. No mention is made of sporotrichosis (other than L42). This problem is a major issue in South America.

Answer: Sporotrichosis is classified as a subcutaneous or implantation mycosis rather than a classical invasive fungal disease. Although disseminated disease may occur in immunocompromised individuals, it is not routinely included within standardized invasive fungal disease definitions used in ECMM/ESCMID or EORTC/MSGERC frameworks. As the survey was structured in accordance with these definitions, sporotrichosis was not a primary focus of the analysis. Nevertheless, in certain regions of South America it represents a significant and, in some settings, increasing public health concern

10. No mention is made of fungal keratitis, which is moderately common and requires samples to be taken and inoculated in clinic by the clinician. Topical natamycin is a WHO Essential Medicine. The authors focus on invasive fungal disease, but blinding infections are a problem too, and the diagnostics are similar.
Answer: The survey was specifically designed to assess diagnostic and therapeutic capacity for invasive fungal diseases. Ophthalmological infections such as fungal keratitis, although clinically important and a recognized cause of visual morbidity in some regions, fall outside the standardized invasive fungal disease frameworks that guided this analysis. Consequently, they were not included within the predefined scope of the survey.

11. There are a large proportion of self citations. Others work should be cited. Examples might include: reference to LAC paracoccidioidomycosis guidelines, PAHO Histoplasmosis guidelines, Asia survey of diagnostics (not by these authors), ATS guidelines on transplant-associated infections and others. This should not be a paper with so many self-citations from Koln.

Answer: We acknowledge the reviewer’s observation regarding citation balance. Several referenced publications represent coordinated regional assessments conducted under a harmonized methodological framework across Latin America, Europe, Africa, and Asia/Pacific; overlapping authorship reflects continuity of this research platform. These studies are necessary to ensure methodological consistency and enable valid cross-regional comparisons rather than preferential citation. Additional regional and international references have been incorporated to ensure balanced contextualization.

We note that several of our self-citations pertain to prior assessments of invasive fungal infection diagnostic and therapeutic capacity across various countries (e.g., Argentina, Peru, Honduras, Brazil, Colombia) and regions (Europe, Asia/Pacific), as well as global guideline development for cryptococcosis, Candida, Mucorales, rare yeasts, rare moulds, and endemic mycoses. These references are critical to understanding the evolution of IFI capacity assessments and provide the foundation for interpreting the current LAC survey results.

We believe that this approach balances methodological continuity with broader literature inclusion, ensuring that readers can appreciate both the historical development of capacity assessments and the current regional landscape.

We thank you for having given us the opportunity of revising and improving the manuscript. Hoping that the revised version of our work might be suitable for publication, we send our best regards.

Sincerely yours,
The authors
